# Immunohistochemical Localization of Key Arachidonic Acid Metabolism Enzymes during Fracture Healing in Mice

**DOI:** 10.1371/journal.pone.0088423

**Published:** 2014-02-07

**Authors:** Hsuan-Ni Lin, J. Patrick O’Connor

**Affiliations:** 1 Department of Biochemistry & Molecular Biology, Graduate School of Biomedical Sciences, Rutgers, The State University of New Jersey, Newark, New Jersey, United States of America; 2 Department of Biochemistry & Molecular Biology, New Jersey Medical School, Rutgers, The State University of New Jersey, Newark, New Jersey, United States of America; Southern Illinois University School of Medicine, United States of America

## Abstract

This study investigated the localization of critical enzymes involved in arachidonic acid metabolism during the initial and regenerative phases of mouse femur fracture healing. Previous studies found that loss of cyclooxygenase-2 activity impairs fracture healing while loss of 5-lipoxygenase activity accelerates healing. These diametric results show that arachidonic acid metabolism has an essential function during fracture healing. To better understand the function of arachidonic acid metabolism during fracture healing, expression of cyclooxygenase-1 (COX-1), cyclooxygenase -2 (COX-2), 5-lipoxygenase (5-LO), and leukotriene A_4_ hydrolase (LTA4H) was localized by immunohistochemistry in time-staged fracture callus specimens. All four enzymes were detected in leukocytes present in the bone marrow and attending inflammatory response that accompanied the fracture. In the tissues surrounding the fracture site, the proportion of leukocytes expressing COX-1, COX-2, or LTA4H decreased while those expressing 5-LO remained high at 4 and 7 days after fracture. This may indicate an inflammation resolution function for 5-LO during fracture healing. Only COX-1 was consistently detected in fracture callus osteoblasts during the later stages of healing (day 14 after fracture). In contrast, callus chondrocytes expressed all four enzymes, though 5-LO appeared to be preferentially expressed in newly differentiated chondrocytes. Most interestingly, osteoclasts consistently and strongly expressed COX-2. In addition to bone surfaces and the growth plate, COX-2 expressing osteoclasts were localized at the chondro-osseous junction of the fracture callus. These observations suggest that arachidonic acid mediated signaling from callus chondrocytes or from callus osteoclasts at the chondro-osseous junction regulate fracture healing.

## Introduction

Genetic ablation or pharmacological inhibition of cyclooxygenase-2 (COX-2) or 5-lipoxygenase (5-LO) significantly affects bone regeneration during fracture healing. Loss of COX-2 activity impairs fracture healing, while loss of 5-LO activity accelerates healing in rodent models [1]–[5]. COX-2 and 5-LO catalyze critical steps in the conversion of arachidonic acid into prostaglandins and leukotrienes, respectively [6], [7]. Prostaglandins and leukotrienes are eicosanoids, which are 20 carbon lipid signaling molecules that regulate many physiological processes including inflammation and pain.

Arachidonic acid metabolism is complex and involves many enzymes, receptors, oxidative mechanisms, and even transcellular synthetic pathways to produce prostaglandins, leukotrienes, lipoxins, resolvins, and other lipid signaling molecules [8]. Prostaglandins and leukotrienes appear to be the most abundant bioactive lipids produced from arachidonic acid. Typically, arachidonic acid is released from cellular membrane stores by the calcium-dependent, cytoplasmic phospholipase (cPLA_2_ or PLA2G4A) and converted into prostaglandin H_2_ (PGH_2_) by cyclooxygenase-1 (COX-1) or COX-2 or into leukotriene A_4_ (LTA_4_) by 5-LO. PGH_2_ and LTA_4_ are intermediates. Specific synthase enzymes convert PGH_2_ into thromboxane A_2_ (TXA_2_), PGD_2_, PGE_2_, PGF_2α_, or PGI_2_ which are then secreted to activate receptors specific for each prostaglandin or thromboxane. Similarly, LTA_4_ is converted into LTB_4_ by leukotriene A_4_ hydrolase (LTA4H) or into LTC_4_ by leukotriene C_4_ synthase. LTC_4_ is further modified to produce the cysteinyl leukotrienes (LTD_4_ and LTE_4_). Typically, these arachidonic acid metabolizing enzymes are abundantly expressed in leukocytes, consistent with the role of prostaglandins and leukotrienes in mediating inflammation [9]. However, these enzymes are expressed in many cell types to mediate other physiological processes [10].

The mechanisms through which COX-2 and 5-LO regulate fracture healing are not understood. In mice lacking COX-2, bone fracture healing is impaired and is characterized by formation of a small, cartilaginous callus [4], [11]. In contrast, fracture healing is accelerated in mice lacking 5-LO in which there is an early, large cartilaginous callus that is rapidly replaced by bone through endochondral ossification [1]. Pharmacological inhibition of COX-2 or 5-LO produces similar effects in rats [2]–[4]. Loss of COX-1 activity does not appear to affect the early stages of healing [4]. Genetic ablation of COX-1, COX-2, or 5-LO alters the levels of PGE_2_, PGF_2α_, and LTB_4_ produced at the fracture site, indicating that these enzymes function at the fracture site [1]. However, which cells express COX-2 or 5-LO during fracture healing has not been determined. Identifying which cells express these enzymes could significantly improve our understanding of the role of COX-2 and 5-LO in bone regeneration.

To address this, we performed an immunohistochemical analysis of time-staged fracture callus specimens from normal mice to identify which cells express COX-1, COX-2, 5-LO, and LTA4H. As expected, all the enzymes localized to leukocytes. We failed to observe consistent expression of these enzymes in osteoblasts. In contrast, COX-1, COX-2, 5-LO, and LTA4H expression was detected in chondrocytes. Interestingly, COX-2 was abundantly expressed in osteoclasts. These data indicate that arachidonic acid metabolism may be regulating the later regenerative stage of fracture healing when endochondral ossification is occurring through an osteoclast, chondrocyte, or osteoclast-chondrocyte mediated mechanism.

## Materials and Methods

### Animal Model

Female ICR mice that were 8–10 weeks old and weighed 28.9±2.6 g (mean ± standard deviation) were used in this study (Taconic Farms, Inc. Germantown, NY). Eight mice were used in each group. Mice were anesthetized by intraperitoneal injection of ketamine and xylazine (0.1 and 0.01 mg/g body weight, respectively). A closed, diaphyseal fracture was created in the right femur using a custom-made, three-point bending device (BBC Specialty Automotive Center, Linden, NJ) as described previously except the mice were allowed to recover for 7 days between insertion of the intramedullary pin and production of the fracture or euthanization [12]. Mice were euthanized at 0 (before fracture but 7 days after intramedullary pin insertion), 6 hours and 1, 2, 4, 7, 10, and 14 days after fracture. Tissues were quickly resected, fixed in Streck Tissue Fixative (Streck, Inc., Omaha, NE) for one day, and then decalcified in 10% EDTA for a week. After decalcification, tissues were embedded in paraffin. All experimental procedures were approved by the University of Medicine and Dentistry of New Jersey-New Jersey Medical School Institutional Animal Care and Use Committee (protocol 10075).

### Histology

Five um thick serial sections were cut, then deparaffinized in three changes of xylene and rehydrated in a graded alcohol series. Osteoclasts were detected by tartrate-resistant acid phosphatase (TRAP) staining as described previously [13]. TRAP stained sections were counterstained with hematoxylin (Sigma-Aldrich). Cartilage was identified by safranin-O staining, then counterstained with Fast Green (Sigma-Aldrich) and hematoxylin. Sections were mounted using Permount mounting medium (Fisher Scientific).

### Immunohistochemistry

COX-1, COX-2, 5-LO, and LTA4H were detected in the paraffin sections by immunohistochemistry using antibodies from Cayman Chemicals (Ann Arbor, MI; rabbit polyclonal anti-COX-1 catalog no. 160109 and rabbit polyclonal anti-COX-2 catalog no. 160126), Cell Signaling Technologies (Danvers, MA; anti-5-LO rabbit monoclonal antibody clone C49G1 catalog no. 3289), and Epitomics, Inc. (Burlingame, CA, anti-LTA4H rabbit monoclonal antibody clone EPR5713, catalog no. 3911-1). For COX-2, antigen retrieval was performed at 70°C for 3 hours in 10 mM sodium citrate buffer, pH 9.5. For COX-1, 5-LO, and LTA4H, antigen retrieval was done at 60°C overnight in 0.2 M boric acid buffer, pH 7.0. After antigen retrieval, the paraffin sections were immersed in SuperBlock buffer (ThermoScientific, Waltham, MA) for 5 minutes to reduce non-specific binding of antibody. Sections were then incubated with primary antibodies overnight at 4°C using an Antibody Amplifier tray (Pro-Histo, LLC, Columbia, SC). Primary antibodies were used at the following dilutions: 1∶700 for anti-COX-1 and for anti-COX-2, 1∶15,000 for anti-LTA4H, and 1∶100 for anti-5-LO and for the rabbit IgG negative control (rabbit DA1E monoclonal IgG, Cell Signaling Technologies). All antibodies were diluted in Amplifying Antibody Dilution Buffer (Pro-Histo, LLC). After incubation with the primary antibody, sections were treated with 3% H_2_O_2_ for 15 minutes to quench endogenous peroxidase activity. Primary antibodies were detected using the Polink-2 Plus HRP with DAB kit (IHC World, LLC, Woodstock, MD), counterstained with methyl green (Sigma-Aldrich), and mounted on glass slides using Permount. Antibody specificity was verified using bone marrow cell extracts prepared from COX-1, COX-2, 5-LO, and LTA4H knockout mice (Figure S1).

### Image Collection and Analysis

Histological specimens were examined using a Nikon Eclipse E800 microscope with a Nikon DS-Fi1 digital camera and Nikon NIS-Elements BR 3.0 imaging software (Nikon Instruments Inc., Melville, NY).

When periosteal leukocytes, periosteal fibroblasts, intramedullary fracture site cells, intramedullary canal cells, external callus leukocytes, or muscle interstitial cells were the target cell populations for counting, five different fields of view were captured as digital images for each specimen using a 20X objective lens. Within each digital image, a 75×75 um (5,625 um^2^) area was selected that contained the appropriate target cell type. The number of target cells and the number of target cells that were positive for enzyme expression as detected by secondary antibody reactivity were manually counted. Counts from the five images collected for each specimen were averaged. For every time point, one specimen (section) from each of the eight mice was examined by immunohistochemistry for each target enzyme. Thus, 4 specimens from each mouse callus were used to detect COX-1, COX-2, 5-LO, and LTA4H, respectively. However, specimens from only 2–8 mice were counted as described above depending upon the presence of the target cell population and quality of the histological specimen. In total, 352 time point, cell type, and target enzyme combinations were examined. Of those combinations, only 2 combinations (Day 4, COX-2, Callus Leukocytes and Day 7, COX-2, Callus Leukocytes) used 2 specimens for cell counting, 5 combinations used 3 specimens, 4 combinations used 4 specimens, 5 combinations used 5 specimens, and the remaining combinations used 6 or more specimens (Tables S3–S13 in File S1). The mean number of enzyme positive cells and total cells were calculated from the counted specimens for each target enzyme and cell type.

When chondrocytes, external callus osteoclasts, or internal callus osteoclasts were the target cell populations for counting, cells were counted as described above except cells were counted within a 200×200 um (40,000 um^2^) area.

When periosteal and endosteal bone lining cells were the target cell population for counting, cells were counted using a set of continuous images captured using the 10X objective lens that covered the entire periosteum or endosteum. Enzyme positive cells, TRAP positive cells, and total cells were identified and counted. The length of the periosteum and endosteum was measured using Image Pro Premier version 9.0 software (Media Cybernetics, Inc., Rockville, MD). Percent positive cells and cells per mm of bone lining were calculated. Data from 2–8 specimens at each time point were averaged. The cell counting methodology is summarized in Table S1 in File S1.

Image collection and cell counting was performed by a single investigator at all times points for all target enzymes. Two additional investigators counted cells for all target enzymes and cell types in the day 7 post-fracture specimens to assess observer-related cell counting variation. No significant differences in cell counts between observers were found and intraclass correlation coefficients were 0.95, 0.87, 0.99, and 0.96 for counting COX-1, COX-2, 5-LO, and LTA4H positive cells, respectively (Figure S2 and Table S2 in File S1). Data were statistically analyzed using SigmaPlot version 12.5 software (Systat Software, Inc. Chicago, IL) or MedCalc version 12.7 software (MedCalc Software bvba, Ostend, Belgium). The statistical analyses are summarized in Tables S3–S23 in File S1.

## Results

### 1. Intact Bone

**Figure 1:**
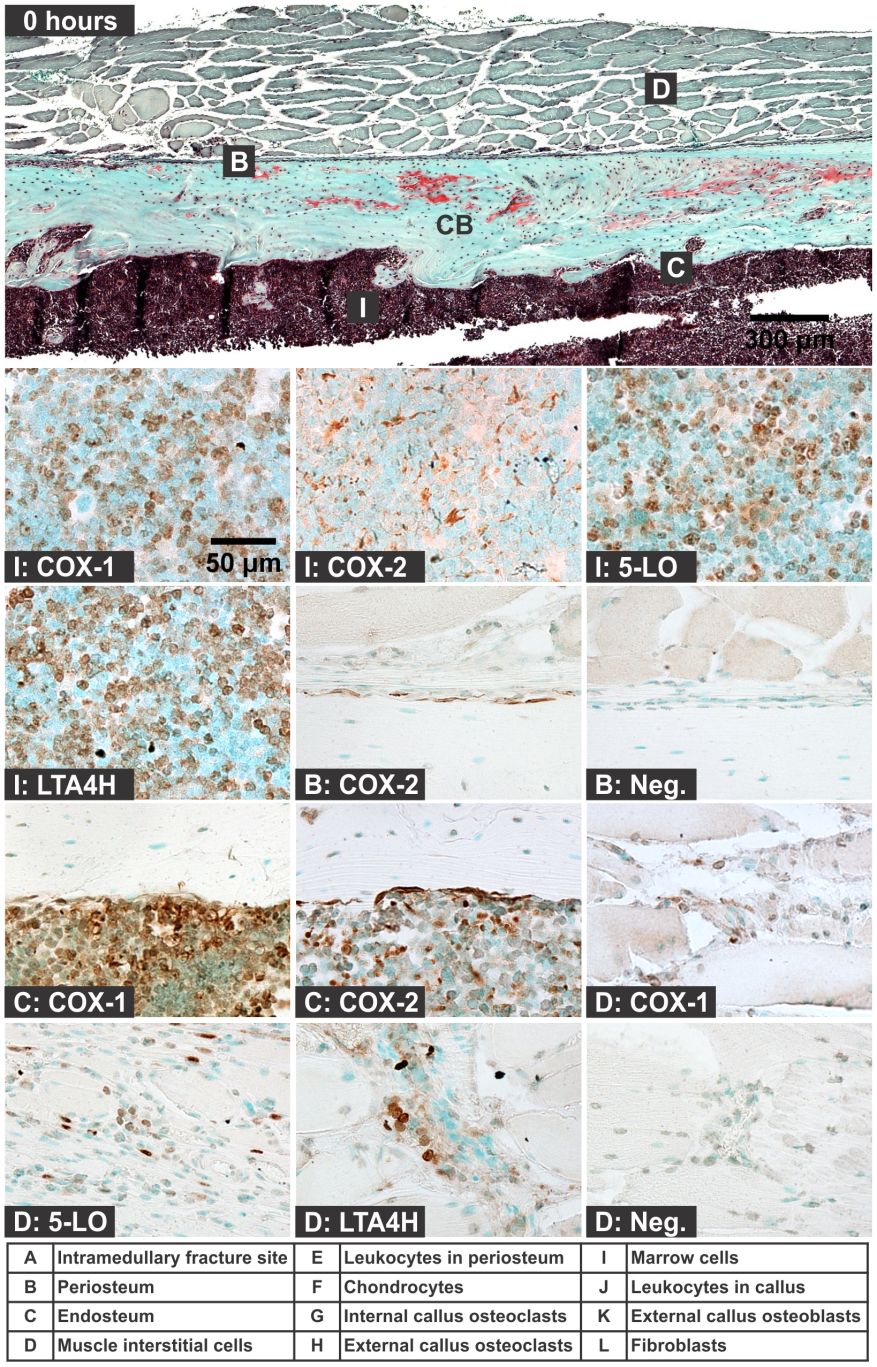
Immunolocalization of Enzyme Positive Cells before Fracture. Top image shows a mouse femur stained with safranin-O (orange) and counter stained with fast green (green) and hematoxylin (black; CB: cortical bone; scale bar: 300 um). Bottom images show immunohistochemical staining of different cell types with antibodies to COX-1, COX-2, 5-LO, or LTA4H (brown; scale bar: 50 um). Rabbit IgG was used as a negative control (Neg.). Immunohistochemistry specimens were counter stained with methyl green. The higher magnification images are labeled with the primary antibody target (COX-1, COX-2, 5-LO, LTA4H, or Neg.) and with a letter indicating cell type and location as listed in the bottom of the figure.

Specimens were collected 7 days after intramedullary rod placement and before any fracture production to assess cellular expression of COX-1, COX-2, 5-LO, and LTA4H in the femur (Figure 1). Cells positive for COX-1, 5-LO, and LTA4H were abundant in the marrow and appeared to be neutrophils based upon morphology (Figure 1I). Cells expressing COX-2 were abundant in the marrow but had a morphology distinct from the COX-1, 5-LO, and LTA4H expressing cells and appeared to be macrophage-like cells (Figure 1I). COX-2 positive cells were present in the periosteum (Figure 1B) and on the endosteal surface (Figure 1C) and appeared flattened, elongated, and associated with sites of bone resorption, suggesting that these COX-2 positive cells are osteoclasts. Some of the endosteal cells also appeared to express COX-1 (Figure 1C). Within the muscle, interstitial leukocytes appeared to be positive for COX-1, 5-LO, and LTA4H (Figure 1D). Endothelial cells did not appear to consistently express 5-LO, though 5-LO positive cells were adjacent to capillaries (Figure 1D). Osteocytes appeared to inconsistently express COX-1 and COX-2 (Figure 1B and 1C).

### 2. Stages of Fracture Healing

In this mouse femur fracture model, healing occurs through 4 temporally and spatially overlapping phases which are the immediate response (6 hours and 1 day after fracture), inflammation (days 1 through 7), regeneration (days 4–21), and remodeling (day 14 onward) [12], [14]. In this model, fractures bridge with new bone at approximately 21 days after fracture. This study focused on the immediate, inflammatory, and regenerative stages of healing and so healing was not assessed after day 14.

**Figure 2:**
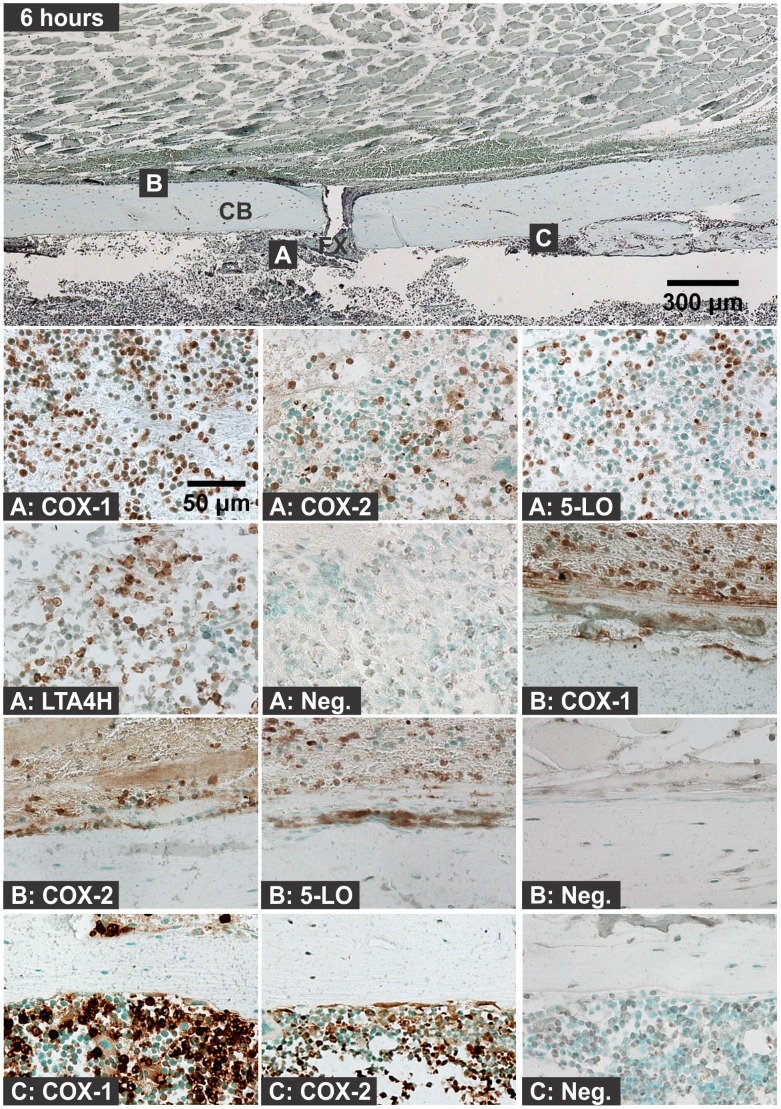
Immunolocalization of Enzyme Positive Cells at 6 hours after Fracture. The top image shows a fractured mouse femur stained with safranin-O (orange) and counter stained with fast green (green) and hematoxylin (black; CB: cortical bone; FX: fracture site; scale bar: 300 um). Bottom images show immunohistochemical staining of different cell types with rabbit IgG (Neg.) or with antibodies to COX-1, COX-2, 5-LO, or LTA4H (brown; scale bar: 50 um). Immunohistochemistry specimens were counter stained with methyl green. The higher magnification images are labeled with the primary antibody target (COX-1, COX-2, 5-LO, LTA4H, or Neg.) and with a letter indicating cell type and location as listed in Figure 1.

**Figure 3:**
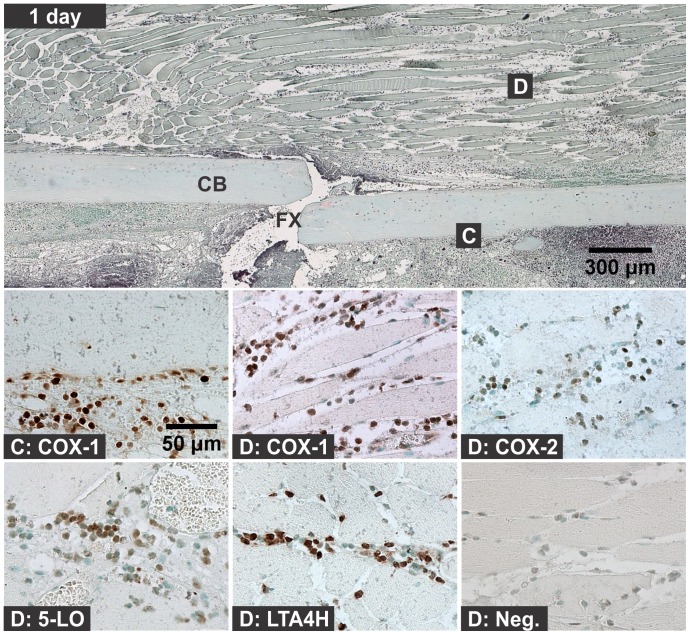
Immunolocalization of Enzyme Positive Cells at 1 day after Fracture. The top image shows a fractured mouse femur stained with safranin-O (orange) and counter stained with fast green (green) and hematoxylin (black; CB: cortical bone; FX: fracture site; scale bar: 300 um). Bottom images show immunohistochemical staining of different cell types with rabbit IgG (Neg.) or with antibodies to COX-1, COX-2, 5-LO, or LTA4H (brown; scale bar: 50 um). Immunohistochemistry specimens were counter stained with methyl green. The higher magnification images are labeled with the primary antibody target (COX-1, COX-2, 5-LO, LTA4H, or Neg.) and with a letter indicating cell type and location as listed in Figure 1.

**Figure 4:**
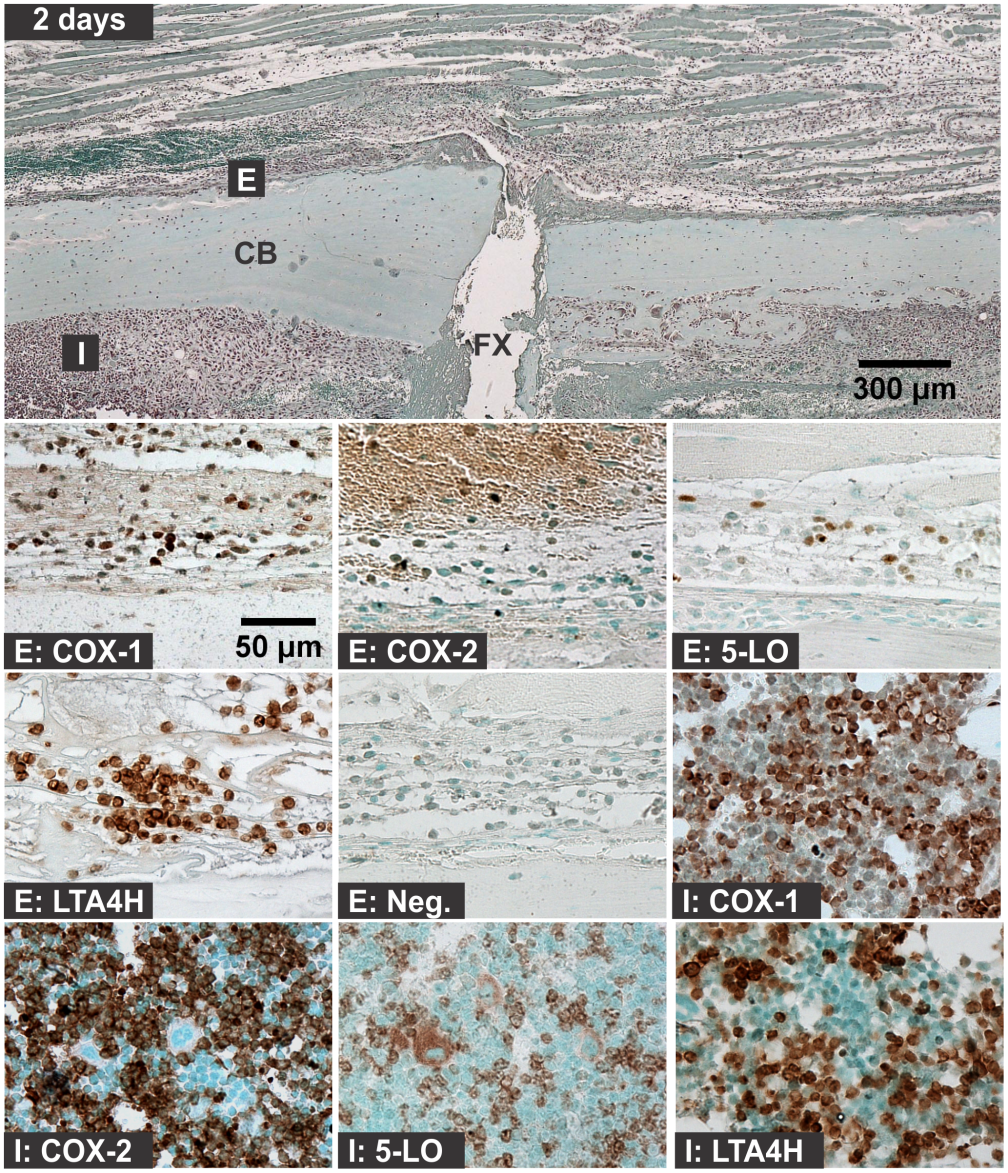
Immunolocalization of Enzyme Positive Cells at 2 days after Fracture. The top image shows a fractured mouse femur stained with safranin-O (orange) and counter stained with fast green (green) and hematoxylin (black; CB: cortical bone; FX: fracture site; scale bar: 300 um). Bottom images show immunohistochemical staining of different cell types with rabbit IgG (Neg.) or with antibodies to COX-1, COX-2, 5-LO, or LTA4H (brown; scale bar: 50 um). Immunohistochemistry specimens were counter stained with methyl green. The higher magnification images are labeled with the primary antibody target (COX-1, COX-2, 5-LO, LTA4H, or Neg.) and with a letter indicating cell type and location as listed in Figure 1.

**Figure 5:**
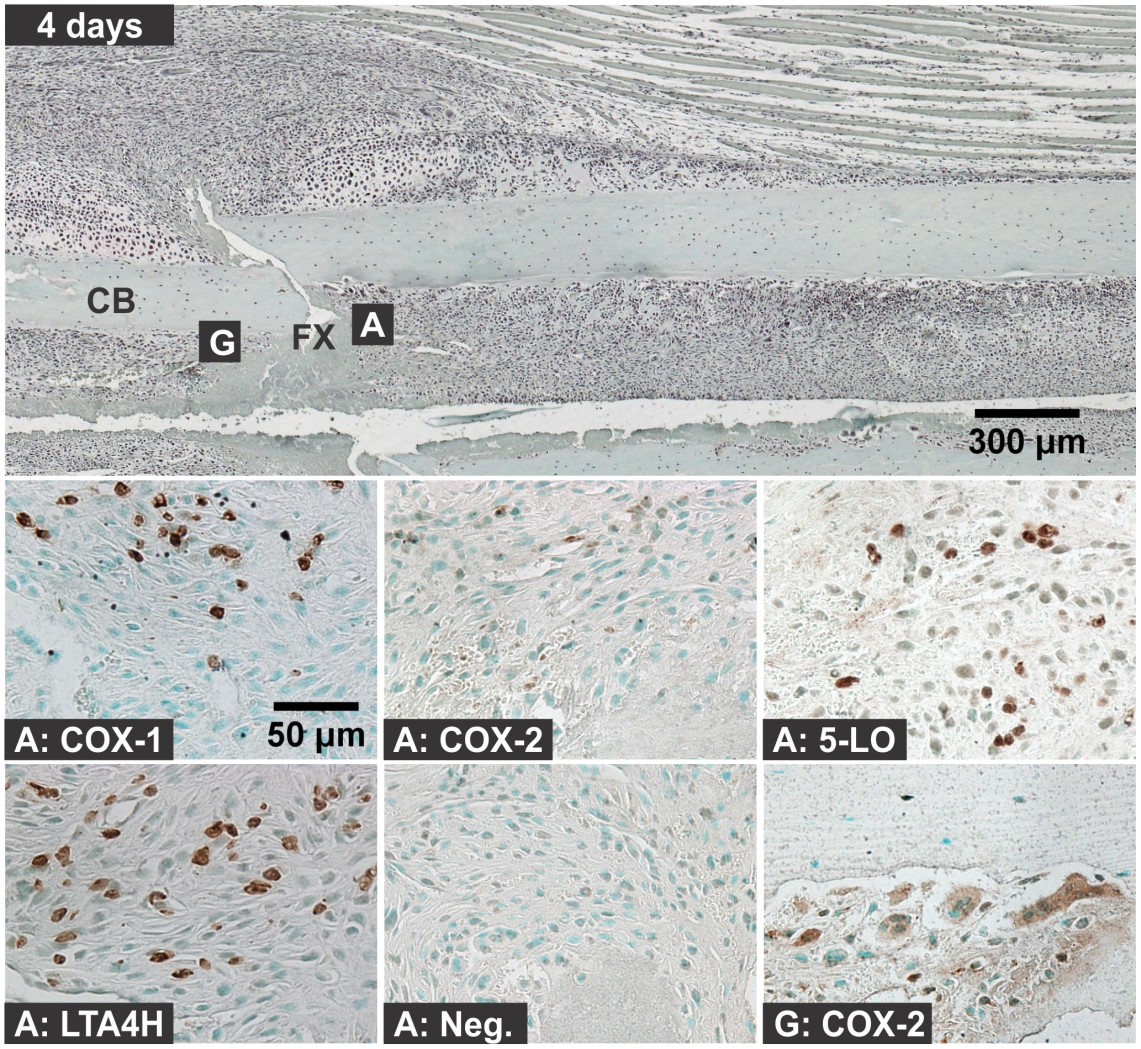
Immunolocalization of Enzyme Positive Cells at 4 days after Fracture. The top image shows a fractured mouse femur stained with safranin-O (orange) and counter stained with fast green (green) and hematoxylin (black; CB: cortical bone; FX: fracture site; scale bar: 300 um). Bottom images show immunohistochemical staining of different cell types with rabbit IgG (Neg.) or with antibodies to COX-1, COX-2, 5-LO, or LTA4H (brown; scale bar: 50 um). Immunohistochemistry specimens were counter stained with methyl green. The higher magnification images are labeled with the primary antibody target (COX-1, COX-2, 5-LO, LTA4H, or Neg.) and with a letter indicating cell type and location as listed in Figure 1.

**Figure 6:**
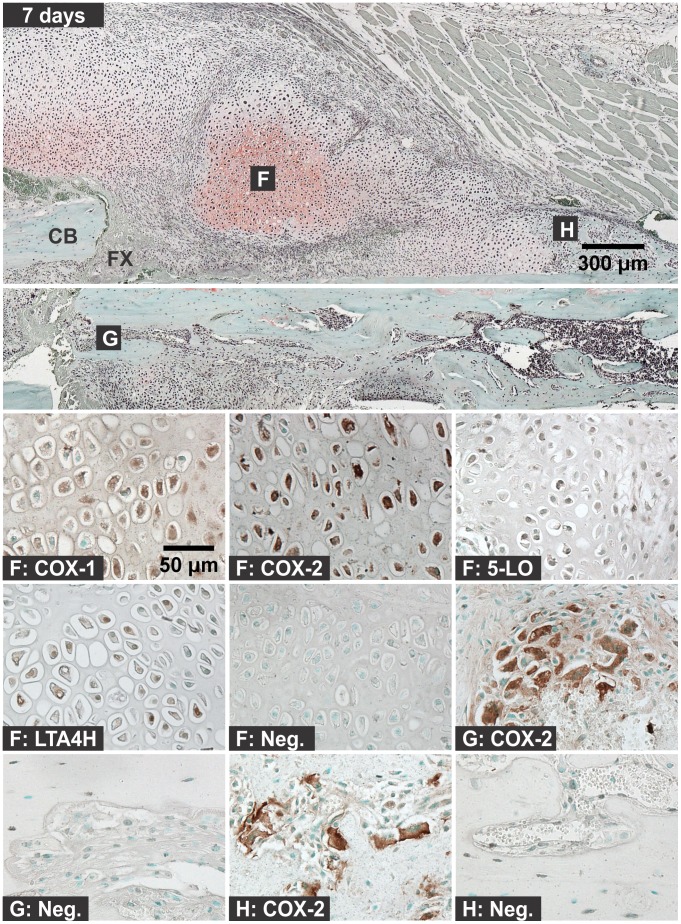
Immunolocalization of Enzyme Positive Cells at 7 days after Fracture. The top image shows the external callus and the image immediately below shows the internal callus of a fractured mouse femur stained with safranin-O (orange) and counter stained with fast green (green) and hematoxylin (black; CB: cortical bone; FX: fracture site; scale bar: 300 um). Bottom images show immunohistochemical staining of different cell types with rabbit IgG (Neg.) or with antibodies to COX-1, COX-2, 5-LO, or LTA4H (brown; scale bar: 50 um). Immunohistochemistry specimens were counter stained with methyl green. The higher magnification images are labeled with the primary antibody target (COX-1, COX-2, 5-LO, LTA4H, or Neg.) and with a letter indicating cell type and location as listed in Figure 1.

**Figure 7:**
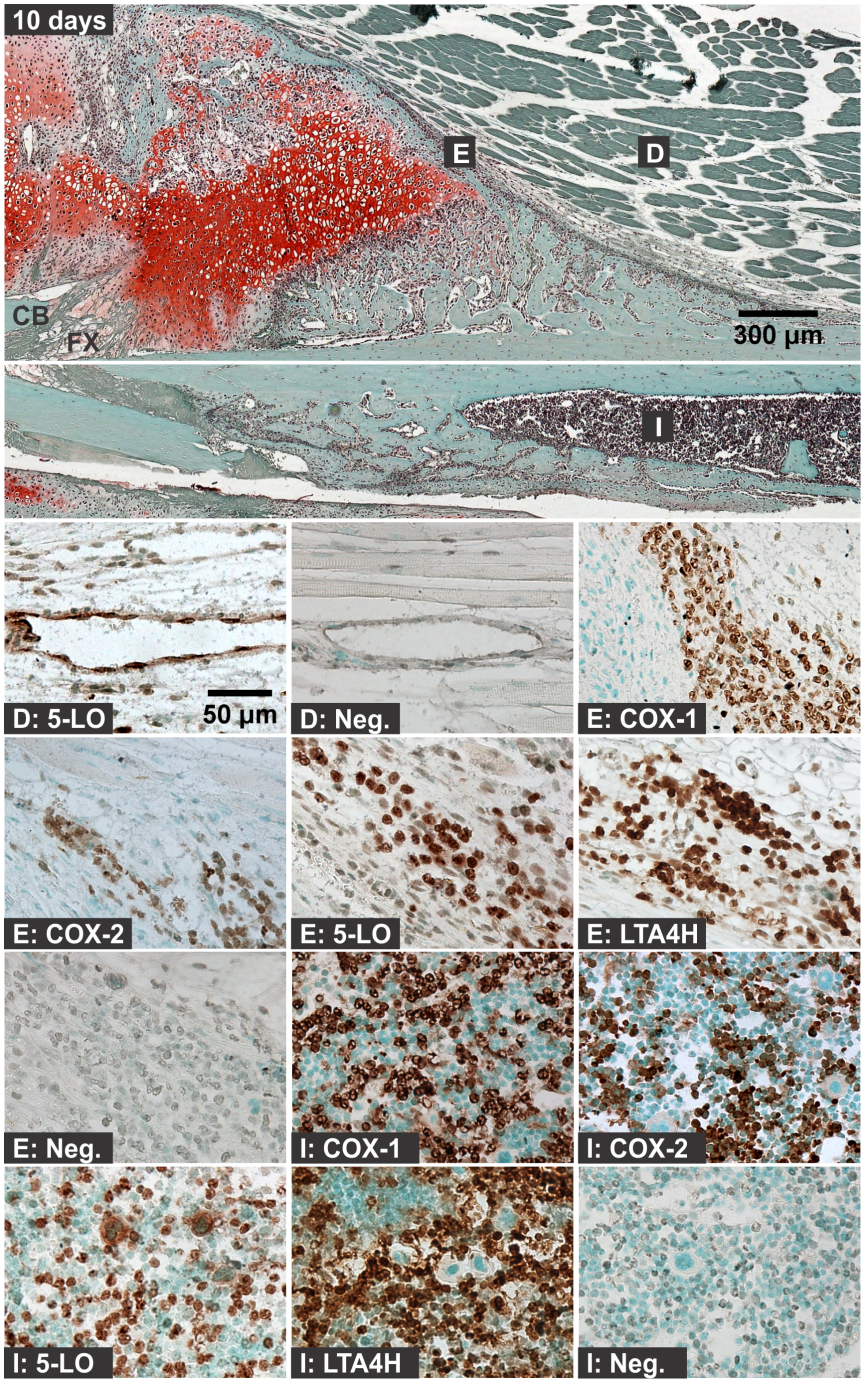
Immunolocalization of Enzyme Positive Cells at 10 days after Fracture. The top image shows the external callus and the image immediately below shows the internal callus of a fractured mouse femur stained with safranin-O (orange) and counter stained with fast green (green) and hematoxylin (black; CB: cortical bone; FX: fracture site; scale bar: 300 um). Bottom images show immunohistochemical staining of different cell types with rabbit IgG (Neg.) or with antibodies to COX-1, COX-2, 5-LO, or LTA4H (brown; scale bar: 50 um). Immunohistochemistry specimens were counter stained with methyl green. The higher magnification images are labeled with the primary antibody target (COX-1, COX-2, 5-LO, LTA4H, or Neg.) and with a letter indicating cell type and location as listed in Figure 1.

**Figure 8:**
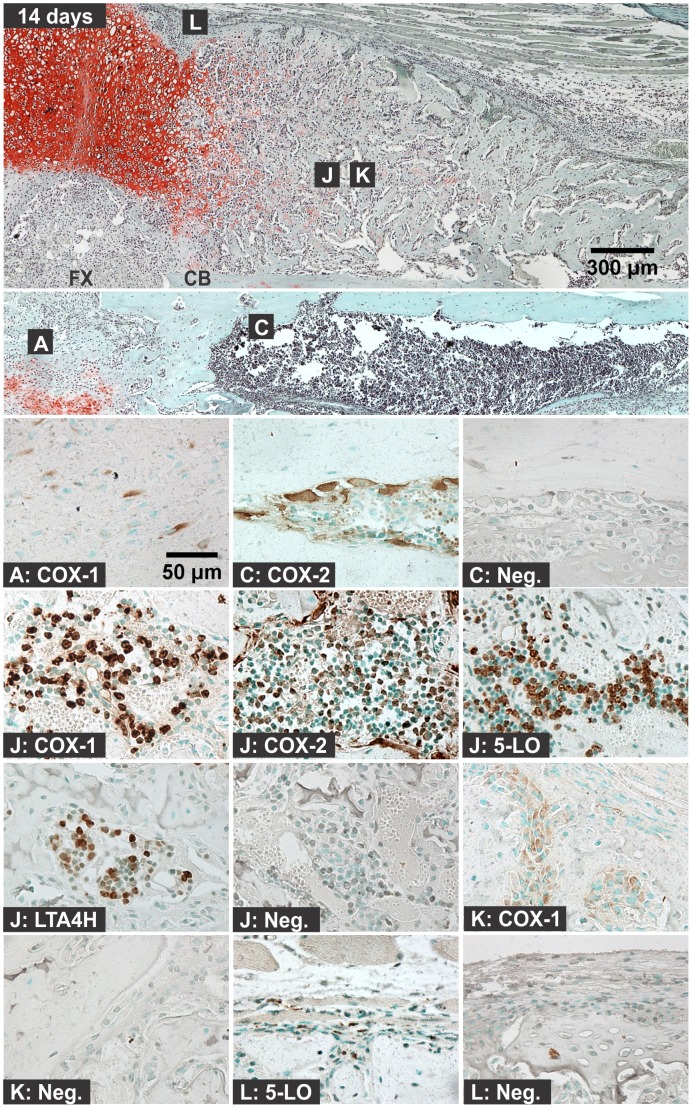
Immunolocalization of Enzyme Positive Cells at 14 days after Fracture. The top image shows the external callus and the image immediately below shows the internal callus of a fractured mouse femur stained with safranin-O (orange) and counter stained with fast green (green) and hematoxylin (black; CB: cortical bone; FX: fracture site; scale bar: 300 um). Bottom images show immunohistochemical staining of different cell types with rabbit IgG (Neg.) or with antibodies to COX-1, COX-2, 5-LO, or LTA4H (brown; scale bar: 50 um). Immunohistochemistry specimens were counter stained with methyl green. The higher magnification images are labeled with the primary antibody target (COX-1, COX-2, 5-LO, LTA4H, or Neg.) and with a letter indicating cell type and location as listed in Figure 1.

Hematoma, leukocytic invasion, tissue swelling, and thickening of the periosteum and endosteum at the fracture site were evident at 6 hours and 1 day after fracture and are consistent with the immediate response to fracture and inflammation initiation (Figures 2 and 3). Mesenchymal cell migration to the intramedullary (internal) and external callus as well as muscle fiber degeneration and more extensive leukocytic infiltration of the soft tissues were evident 2 days after fracture (Figure 4). By 4 days after fracture, a distinct, external callus morphology was evident which included newly formed periosteal bone at the periphery of the fracture callus, mesenchymal cells at the center of the callus, and chondrocytes between the newly formed bone and centrally located mesenchymal cells (Figure 5). Apparent endochondral ossification was evident at days 7, 10, and 14 after fracture and was associated with increased callus size that appeared to peak at day 10, cartilage formation based upon safranin-O staining, and bone formation from the peripheral edges of the callus towards the centrally located fracture site (Figures 6–8). Also evident was reduced swelling, intact muscle fibers, and reduced leukocytic infiltration as healing progressed.

### 3. Cell-Type Specific Expression

The expression of COX-1, COX-2, 5-LO, and LTA4H in the major cell types present during fracture healing are described below.

#### 3a: Leukocytes

Inflammation is mediated by leukocytes and bone marrow is rich with leukocytes. In this study, we did not attempt to distinguish different populations of leukocytes using immunohistochemical methods. Cells were considered leukocytes based upon their morphology and locations within the tissues.

**Figure 9:**
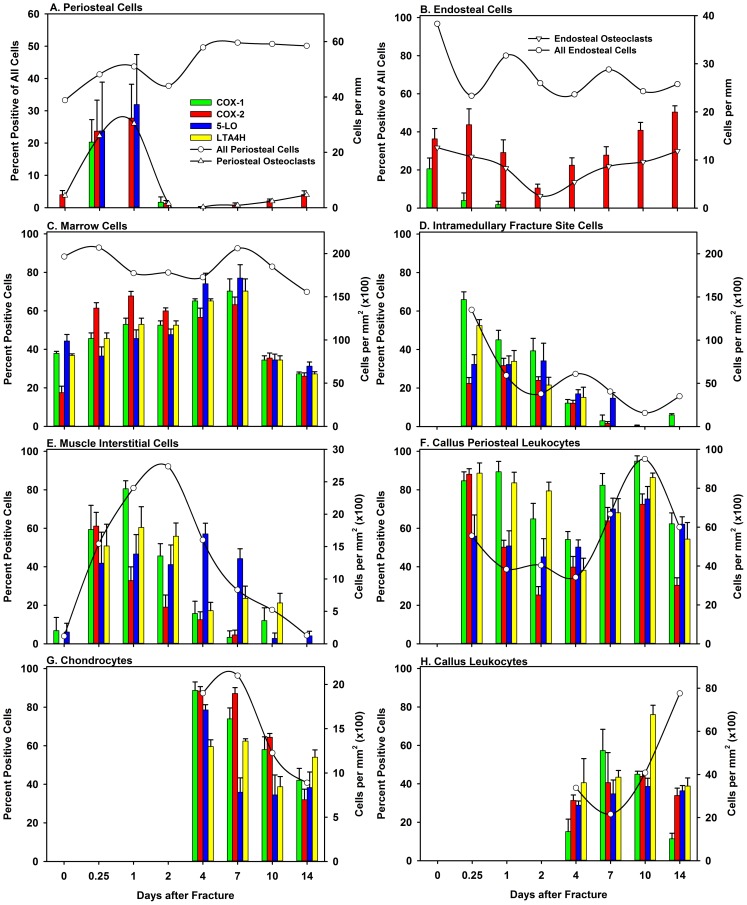
Quantitation of Enzyme Positive Cells during Fracture Healing. The total number of each cell type was counted from before till 14 days after fracture as described in the Materials and Methods and are shown as cells per mm or cells per mm^2^ (circles; right axis). The number of periosteal (triangles, right axis) and endosteal (inverted triangles, right axis) osteoclasts were also counted (Panels A and B, respectively). The percentage of cells positive for COX-1 (green), COX-2 (red), 5-LO (blue), or LTA4H (yellow) was calculated and mean values (+S.E.M) are shown (left axis). Cell types are indicated for each panel. For Panel D: Intramedullary Fracture Site Cells and Panel F: Callus Periosteal Leukocytes, no data were available before fracture. For Panel G: Chondrocytes and Panel H: Callus Leukocytes, no chondrocytes or external callus marrow containing leukocytes were present prior to day 4 after fracture and so no values were entered for earlier time points. Note that the X-axis scales (Days after Fracture) are not linear with respect to time.

Following fracture, leukocytes were evident in the marrow of the intramedullary canal and external fracture callus, the periosteum, and surrounding muscle at the fracture site. The number of leukocytes in the surrounding muscle increased and decreased in a pattern consistent with inflammation amplification and resolution (Figure 9E). The leukocytes were positive for COX-1, COX-2, 5-LO, or LTA4H at all sites and times after fracture. Further, leukocytes present in the bone marrow at a significant distance from the fracture site were positive for COX-1, COX-2, 5-LO, or LTA4H suggesting that these enzymes are normally expressed in these cells (Figures 1I, 4I and 7I).

After fracture, the periosteum typically thickens into multiple cell layers near the fracture site as osteoprogenitor cells within the periosteum proliferate [15], [16]. The periosteum also appeared to be invaded by leukocytes that were positive for COX-1, COX-2, 5-LO, or LTA4H expression (Figure 4E). As healing progressed, the periosteum appeared as a layer of cells between the external surface of the callus and the surrounding muscle. Leukocytes positive for COX-1, COX-2, 5-LO, or LTA4H expression were present within the callus periosteum 14 days after fracture, even though inflammation had subsided and significant amounts of cartilage and bone had already formed (Figures 7E, 8, and 9F).

#### 3b: Osteoblasts

For these experiments, osteoblasts were identified by morphology and localization next to bone. At one day after fracture, some endosteal cells with the appearance of osteoblasts were positive for COX-1 in one of six specimens (Figure 3C). Additional COX-1 expression was not observed in osteoblasts until day 14 after fracture. In all the 14 day fracture callus specimens, COX-1 expression was observed in some of the external callus osteoblasts (Figure 8K). COX-2 expression was observed in endosteal, periosteal, and external fracture callus osteoblasts at different time points (Figure 2B). However, the staining was faint compared to other COX-2 positive cell types and was not consistently observed between specimens. Expression of 5-LO and LTA4H was not observed in osteoblasts.

#### 3c: Chondrocytes

For these experiments, chondrocytes were identified based upon morphology, position within the fracture callus, and staining with safranin-O. Chondrocytes were evident by day 4 after fracture (Figure 5). Chondrocytes positive for COX-1, COX-2, 5-LO, or LTA4H were observed at 4, 7, 10, and 14 days after fracture (Figure 6F). The percentage of COX-1, COX-2, 5-LO, or LTA4H positive chondrocytes decreased as healing progressed (Figure 9G). However, there was a sharp decline in the proportion of 5-LO positive chondrocytes between days 4 (79%) and 7 (40%; Figure 9G).

#### 3d: Osteoclasts

Osteoclasts were identified based upon morphology and localization on mineralized tissue surface. In early time points after fracture (6 hours to 2 days), cells on the bone surface with morphology similar to osteoclasts or osteal macrophages were positive for COX-1, COX-2, or 5-LO, but not for LTA4H (Figures 2B and 2C) [17]. At all subsequent time points, osteoclasts were negative for COX-1, 5-LO, and LTA4H. Osteoclasts positive for COX-2 expression were observed at all time points and locations within the fracture callus and growth plate (data not shown). COX-2 positive osteoclasts were readily observed in the intramedullary canal at the fracture site by 4 days after fracture and in the external callus by 7 days after fracture (Figures 5G, 6G, 6H, and 8C).

**Figure 10:**
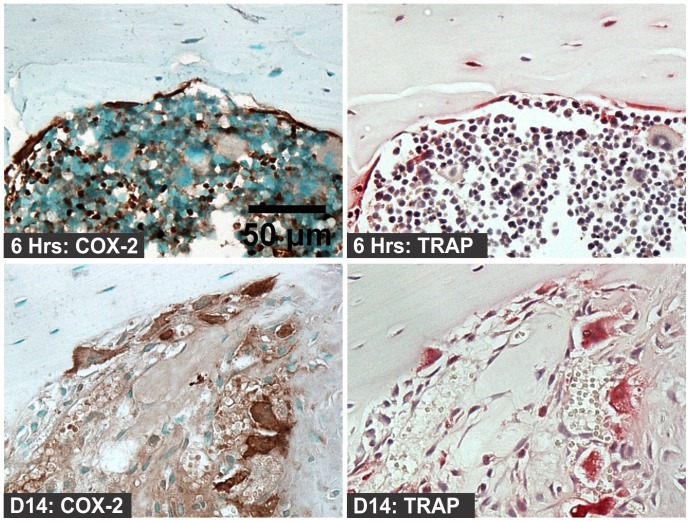
Co-localization of TRAP and COX-2 Positive Cells. Serial sections from mouse femurs at 6 hours (top images) and 14 days (bottom images) after fracture were used for immunohistochemical detection of COX-2 expressing cells (left images; brown staining) and for tartrate-resistant acid phosphatase staining (right images; red staining; scale bar: 50 um).

**Figure 11:**
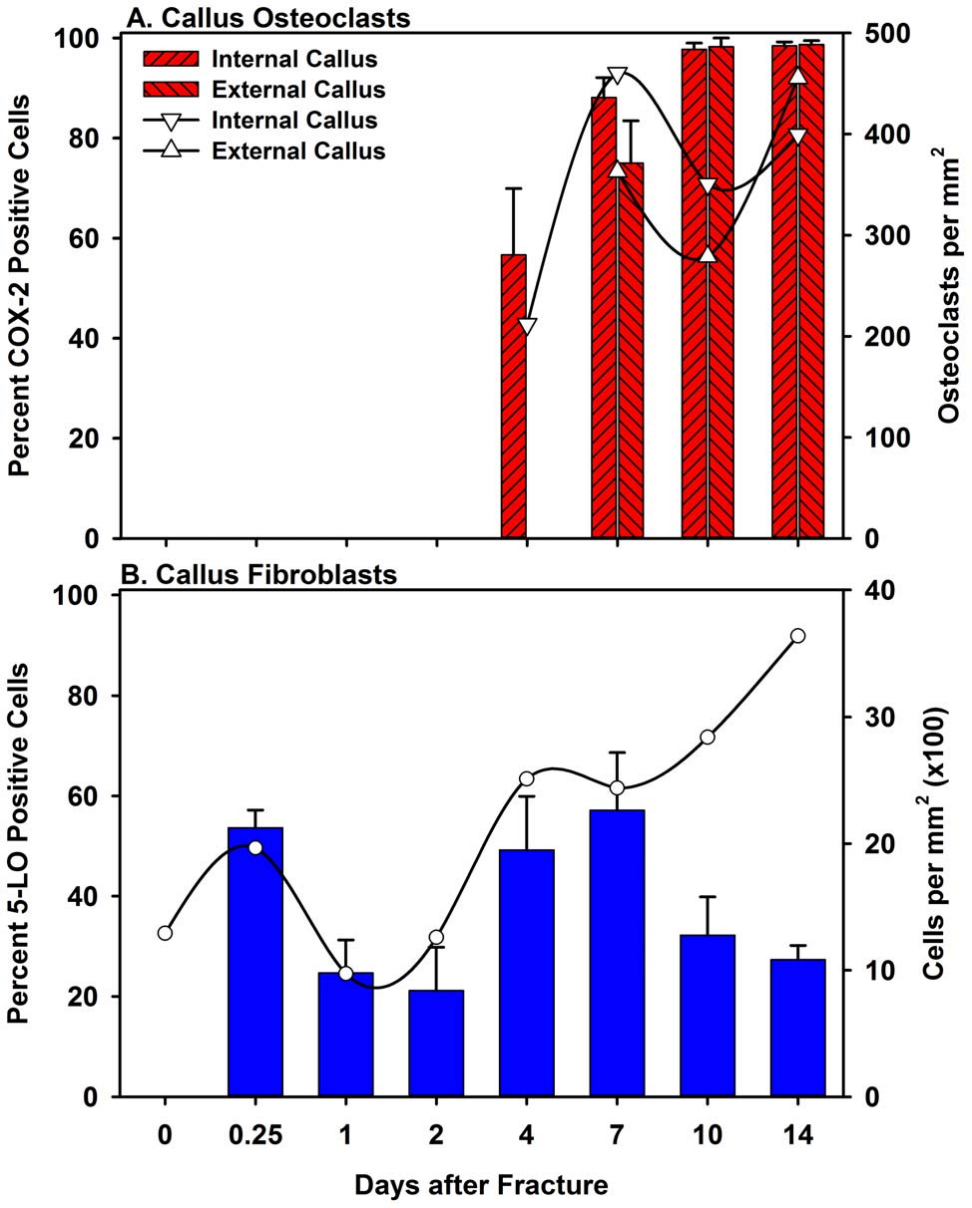
Quantitative Analysis of Fracture Site COX-2 Positive Osteoclasts and 5-LO Positive Fibroblasts. The number of external callus osteoclast (triangles), internal callus osteoclast (inverted triangles), and callus fibroblasts (circles) were counted (right axis). The percentage of osteoclast that also were positive for COX-2 by immunohistochemical detection were calculated for the external (right bars)and internal callus (left bars) at each time point and are shown as mean values (+ S.E.M.; Panel A; left axis). The percentage of fibroblasts positive for 5-LO by immunohistochemistry were calculated at each time point and are shown as mean values (+S.E.M.; Panel B; left axis). Note that the X-axis scales (Days after Fracture) are not linear with respect to time.

To confirm that these COX-2 positive cells were indeed osteoclasts, serial sections were examined for COX-2 expression by immunohistochemistry and for tartrate-resistance acid phosphatase (TRAP) activity by histochemical staining. As shown in Figure 10, COX-2 positive cells on the bone surface were also TRAP positive. In the internal and external callus, the percentage of osteoclasts that were COX-2 positive varied as healing proceeded (Figure 11A). In the internal callus, the percentage of TRAP positive cells that were also COX-2 positive increased from about 55% on day 4 to almost 100% on day 14. In the external callus, the number of COX-2 positive TRAP cells increased from 75% on day 7 to almost 100% on day 14.

#### 3e: Endothelial cells

Angiogenesis is essential for fracture healing and capillaries were evident in the fracture callus and surrounding muscle. The endothelial cells of the capillaries were positive for 5-LO expression from day 7 onward (Figure 7D). Endothelial cells positive for COX-1 were also observed in one of six samples on day 4 (not shown).

#### 3f: Fibroblasts

Some spindle-shaped cells in the periosteum were positive for 5-LO expression at all time points after fracture (Figure 8L). Approximately 50% of these callus fibroblasts were positive for 5-LO at 6 hours and 4 and 7 days after fracture with lesser amounts at days 1,2, 10, and 14 (Figure 11B).

### 4. The Chondro-Osseous Junction

**Figure 12:**
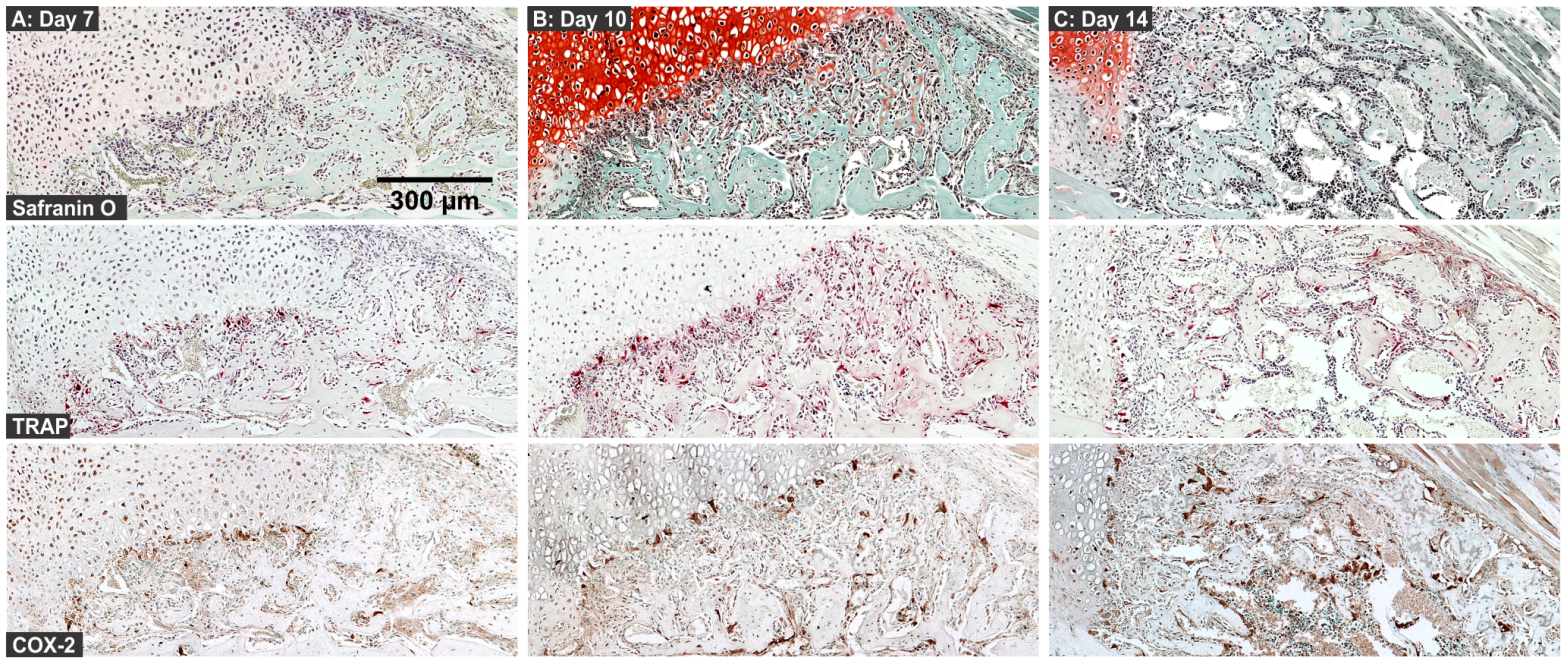
Osteoclasts at the Chondro-Osseous Junction during Fracture Healing. Serial sections from mouse femur fractures collected at 7 (A), 10 (B), and 14 days (C) after fracture were stained with safranin-O (top images), TRAP (middle images), or stained for COX-2 using immunohistochemistry (bottom images; scale bar: 300 um). The images show that COX-2 expressing osteoclasts appear to preferentially localize at the junction between callus cartilage and the zone of active new bone formation during fracture healing.

A striking feature of fracture callus morphology and COX-2 expression is shown in Figure 12. The interface between the cartilage stained with safranin-O and the zone of calcified cartilage and bone formation was highly enriched with cells that co-express TRAP and COX-2 and appeared to be osteoclasts.

### 5. Quantitative Analysis of Enzyme Positive Cells

The percentage of enzyme positive cells within different regions of the callus was determined. The total number of cells with the same morphology was also counted in that tissue region to derive the proportion of enzyme positive cells. The data are summarized in Figures 9 and 11. These data were analyzed using ANOVA and post-hoc Holm-Sidak tests to identify differences in the percent of enzyme positive cells with time after fracture (Tables S3–S13 in File S1). The percentage of periosteal cells that expressed COX-1, COX-2, or 5-LO peaked by day 1 after fracture before rapidly declining (Figure 9A). In contrast, endosteal cells preferentially expressed COX-2 throughout healing (Figure 9B). The COX-2 positive bone lining cells appeared to be osteoclasts and the number of apparent osteoclasts (TRAP positive cells identified in serial sections) was highly correlated to the number of COX-2 positive cells on the periosteal (R = 0.997) and endosteal surfaces (R = 0.916) using a Gaussian peak fit curve. The total number of muscle interstitial cells peaked at day 2 after fracture and showed an early preference for expressing COX-1 and COX-2 and a later preference for expressing 5-LO (Figure 9E). Conversely, 5-LO expression in chondrocytes occurred early while COX-1 and COX-2 expression continued into later times (Figure 9G). Callus periosteal leukocytes peaked at day 10 after fracture, which coincides with peak callus volume (Figure 9F). Osteoclasts were observed at the intramedullary fracture site by day 4 after fracture and in the external callus by day 7 (Figure 11A). By day 10 after fracture, over 95% of callus osteoclasts were positive for COX-2 and negative for the other target enzymes.

## Discussion

We performed an immunohistochemical analysis of time-staged mouse fracture callus specimens to identify cells that express COX-1, COX-2, 5-LO, and LTA4H with the goal of identifying potential mechanisms through which arachidonic acid metabolism regulates fracture healing. We used morphological observations to identify cell types rather than performing additional experiments to confirm each cell type except for using TRAP or safranin-O staining to aid in identifying osteoclasts and chondrocytes, respectively. This is a limitation of the study particularly with the leukocytes as we cannot precisely assign enzyme expression to specific leukocyte types, such as neutrophils, T-cells, or macrophages. Additional experiments will be needed to refine assignment of COX-1, COX-2, 5-LO, or LTA4H expression to cell type subpopulations. It also should be noted that expression of COX-1, COX-2, 5-LO, or LTA4H does not equate with enzyme activity. Rather the presence of these enzymes indicates that a cell has the potential capacity to produce prostaglandins or leukotrienes when appropriately stimulated.

As expected, COX-1, COX-2, 5-LO, and LTA4H were expressed in multiple cell types over the time course examined. Enzyme positive leukocytes increased at the fracture site and surrounding muscle during the early stages of healing and then decreased. Osteoblasts in the periosteum and in the newly formed bone of the callus did not appear to express COX-2, 5-LO, or LTA4H to an extent that could be reliably detected using these immunohistochemical methods. COX-1 expression was detected in osteoblasts of newly formed callus bone. This was unexpected since COX-1 and COX-2 expression had been detected in rat tibia osteoblasts and induction of COX-2 expression in cultured osteoblasts has been repeatedly observed [18]–[24]. In contrast, callus chondrocytes expressed COX-1, COX-2, 5-LO, and LTA4H. Osteoclasts and osteal macrophages appeared to abundantly express COX-2 and transiently express COX-1 and 5-LO during the early stages of healing (6 hours, 1, and 2 days). Previous studies found that COX-2 mRNA levels were elevated immediately after fracture during the inflammation phase, declined, and then peaked again [2], [25]. The basis for the second increase in COX-2 expression was never fully explained. However, the data presented here indicate that the second peak in COX-2 mRNA correlates with COX-2 expression in the callus chondrocytes and an abundance of COX-2 positive osteoclasts present in the callus during endochondral ossification.

The continued presence of enzyme positive leukocytes and 5-LO positive fibroblasts at the interface of the callus and surrounding muscle (callus periosteum), even at the later stages of healing suggests that these 5-LO positive cells could be secreting eicosanoids to regulate the healing process. In support of this concept, chondrocytes can utilize leukotriene A_4_ synthesized from polymorphonuclear leukocytes to produce leukotriene B_4_ and C_4_ through a transcellular process [26]. The 5-LO positive cells also could be aiding in additional functions known to be mediated by 5-LO, including angiogenesis and the resolution of inflammation [28]–[31].

Osteoblasts are necessary for bone formation and consequently for fracture healing. Numerous studies have demonstrated that COX-2 expression can be induced in cultured osteoblasts [18], [19], [22], [23], [32]–[37]. Many other studies have shown that COX-2 activity in cultured osteoblasts or prostaglandin treatment of osteoblasts can promote proliferation or differentiation [38]–[44]. In vivo, treatment with prostaglandins or prostaglandin analogs can promote bone formation and enhance fracture healing [45]–[49]. In contrast, leukotrienes can inhibit osteoblast proliferation and impair bone formation [50], [51]. In vivo, COX-1 and COX-2 expression was previously detected in endosteal osteoblasts and cortical bone osteocytes of the rat tibiae with little apparent expression of either enzyme in periosteal osteoblasts [24]. In this study, COX-2 was not consistently detected in bone-surface osteoblasts (Figure 2B). A previous study concluded that fracture healing is impaired in COX-2 null mice because loss of COX-2 function impairs osteoblast differentiation in vitro [11]. However, lack of COX-2 expression in osteoblasts in vivo, as shown here, and the ability of COX-2 null, as well COX-1, 5-LO, or LTA4H null mice to develop normally sized and growing skeletons indicates that expression of these enzymes in osteoblasts is not essential for osteoblast function [52]–[54]. Indeed, a femur segment from a wild-type mouse transplanted into a COX-2 null mouse fails to heal while a femur segment from a COX-2 null mouse transplanted into a wild-type mouse does heal indicating that COX-2 null osteoblasts can participate in healing within a COX-2 sufficient environment [55]. We suggest that eicosanoids synthesized by leukocytes, chondrocytes, and osteoclasts that are in close proximity to osteoblasts and mesenchymal cells during fracture healing may be regulating the proliferation and differentiation of osteoblasts through cell dependent signaling. Such a mechanism could be mediated by osteal macrophages (osteomacs) that organize as a cellular network or canopy over bone surface osteoblasts [17]. While it is possible that expression of COX-1, COX-2, 5-LO, or LTA4H was not consistently detected in osteoblasts for technical reasons, the positive expression of the target enzymes in other cell types of the same specimen indicates that this possibility is unlikely.

The significance of the COX-1, COX-2, 5-LO, and LTA4H expression in the callus chondrocytes is not yet known. Expression of these enzymes in growth plate and in normal or pathological articular chondrocytes has been detected previously [26], [56]–[59]. In rat tibia growth plates, COX-1 was strongly expressed in the reserve zone, while only moderate COX-2 expression was detected in the reserve zone [57]. In cultured rat growth plate chondrocytes, cell proliferation was inhibited by treatment with SC-236, a COX-2 selective inhibitor, but not with SC-560, a COX-1 selective inhibitor [57]. Proliferation of the cultured rat growth plate chondrocytes could be stimulated with PGE_2_ or with a selective EP1 agonists [57]. Consistent with a COX-2 proliferative effect on growth plate chondrocytes, treatment of chicken growth plate chondrocytes with PGE_2_ inhibited Type X collagen, VEGF, and MMP-13 expression indicating that PGE_2_ treatment was inhibiting chondrocyte differentiation [60]. Similarly, treatment of osteoarthritic chondrocytes with butaprost, a PGE_2_ analog that specifically activates the EP2 receptor, also reduced MMP-13 expression [61]. Less is known of leukotriene effects on chondrocyte functions. Treatment of articular chondrocytes with IL-1ß reduced synthesis of LTB_4_ while inducing synthesis of PGE_2_ [62]. In contrast, treatment of human osteoarthritic articular chondrocytes with naproxen, a non-selective COX-1 and COX-2 inhibitor, caused a significant increase in chondrocyte LTB_4_ synthesis [59]. Expression of COX-2 can be induced in articular chondrocytes by treatment with IL-1ß while expression of 5-LO and its essential co-factor five lipoxygenase activating protein (FLAP) can be induced with TGF-ß and 1, 25-dihydroxy vitamin D_3_ [56], [59]. In fracture calluses, inhibition of 5-LO appears to accelerate chondrocyte differentiation during endochondral ossification and increases COX-2 expression, while inhibition of COX-2 appears to prevent chondrocyte differentiation leading to impaired endochondral ossification [2]. These prior observation show that chondrocyte proliferation and differentiation are affected by prostaglandins. In addition, chondrocytes can interact with other cell types to produce leukotrienes through transcellular pathways [26]. However, additional experiments are needed to determine whether callus chondrocytes behave similarly to growth plate or articular chondrocytes with respect to eicosanoid treatment, whether callus chondrocyte expression of these enzymes signifies a response to the local growth factor milieu, or whether callus chondrocyte expression of these enzymes underlies a regulatory role for chondrocytes in the regenerative process.

Of the 4 enzymes, only COX-2 expression was detected consistently in osteoclasts at the fracture site. Osteoclasts within the growth plate of the fractured femurs or in control, unfractured femurs were also positive for COX-2 (data not shown) and immunohistochemical detection of COX-2 expression was severely reduced in mice in which COX-2 was deleted in osteoclasts (Figure S3). A previous report also demonstrated expression of COX-2 in osteoclasts by immunohistochemical detection in rat tibiae [24]. Not all the TRAP and COX-2 positive cells appeared to be multinucleated in our specimens and some of the COX-2 and TRAP positive cells may be macrophages, dendritic cells, monocytes differentiating into osteoclasts, or macrophages or dendritic cells transdifferentiating into osteoclasts [63]–[67]. The proportion of TRAP positive cells in the fracture callus that were also COX-2 positive increased to almost 100% over time (Figure 11A). This suggests that either a population of TRAP positive, COX-2 negative cells are present early in healing and then disappear or that COX-2 expression increases as TRAP positive cells mature into osteoclasts.

The importance of COX-2 in osteoclast biology is well established [68]–[71]. Stimuli that induce COX-2 expression and activity in osteoblasts or bone marrow cells in turn enable the osteoblasts or bone marrow cells to promote osteoclast differentiation [33]. Thus, COX-2 has been thought to regulate osteoclast biology only through the activity of other cell types. However, the clear demonstration that COX-2 is expressed in osteoclasts themselves suggests that COX-2 may have a cell-autonomous function in osteoclasts. One role may be directly related to osteoclast development since prostaglandins produced by osteoclasts may aid in osteoclast formation and function through an autocrine signaling mechanism. Spleen or bone marrow cells from COX-2 null mice have a reduced capacity to form osteoclasts which can be rescued by exogenous PGE_2_ treatment [68]. Further, COX-2 expression can be induced by a variety of stimuli including RANKL in osteoclast precursors such as monocytes or macrophage-like cell lines that can be induced to differentiate into osteoclasts [70], [72], [73]. This suggests a direct role for COX-2 in osteoclastogenesis in which stimuli, such as, RANKL induces COX-2 expression in osteoclast precursors and the prostaglandins produced by that COX-2 act in an autocrine feedback mechanism to promote osteoclastogenesis.

Parallel observations between effects of impaired osteoclast activity and impaired COX-2 activity indicate that osteoclast COX-2 activity may be regulating fracture healing. The localization of COX-2 positive osteoclasts at the interface of callus cartilage and active endochondral ossification (chondro-osseous junction) suggests that the osteoclasts may be the critical regulatory cell controlling the regenerative phase of fracture healing (Figure 12). Classically, these osteoclasts have also been referred to as chondroclasts [74]. Osteoclast activity is necessary to resorb the calcified cartilage produced by the hypertrophic chondrocytes and to provide calcified tissue surfaces for new bone formation [75]–[77]. Osteoclasts may also promote chondrocyte hypertrophy and calcified cartilage formation necessary for endochondral ossification to proceed. In mice treated with zoledronate to inhibit osteoclast activity, expression of Type X collagen was delayed in tibia fracture calluses [78]. Similarly, in rats treated with celecoxib to inhibit COX-2 activity, fracture callus Type X collagen expression was significantly reduced [2]. Finally, macrophage-like cells present in the periosteum (osteomacs) which could potentially differentiate into osteoclasts have been shown to enhance osteoblast activity and bone formation [17]. Indeed, prostaglandins produced by COX-2 can promote osteoblast activity [78]. Together, the observations support a model in which COX-2 exerts its regulatory functions on fracture healing through the osteoclast.

## Supporting Information

Figure S1 Antibody Specificity. The specificity of the antibodies used to detect COX-1, COX-2, 5-LO, and LTA4H was verified be using bone marrow derived cells and macrophages. Total protein extracts prepared from the bone marrow of COX-1, COX-2, 5-LO, and LTA4H knockout mice were analyzed for COX-1, 5-LO, and LTA4H expression by immunoblot analysis (Bone Marrow Cells). Expression of ß-tubulin was used as a control. The antibodies for COX-1, 5-LO, and LTA4H failed to detect any protein in their corresponding knockout bone marrow cell extracts but did detect proteins of the correct size (markers not shown) in the other bone marrow cell extracts. To detect COX-2 expression, bone marrow cells from wild-type and COX-2 knockout mice were cultured in DMEM with 10% FBS and 20% L929 conditioned media to promote macrophage development (Macrophages). The cultures were then induced to express COX-2 by treating with 100 ng/ml of LPS overnight followed by protein extract preparation and immunoblot analysis for COX-2 expression. The COX-2 antibody detected a protein of the correct size in the LPS-treated wild-type cells but failed to detect any protein in the COX-2 knockout cells. Antibodies were detected by chemiluminescence using appropriate horseradish peroxidase conjugated secondary antibodies and a Proteinsimple Fluorchem M imaging system.
(TIF)

Figure S2 Inter-Observer Variation for Each Antibody. Consistency between observers for the percentage of antibody positive cells counted in each sample was determined using intraclass correlation coefficient tests. The cumulative frequency distribution for each observer (or rater) and each antibody (A: COX-1, B: COX-2, C: 5-LO, and D: LTA4H) are shown with maximum 100% positive target cells.
(TIF)

Figure S3 Expression of COX-2 in Osteoclasts. Mice homozygous for a floxed allele of COX-2 (COX-2 f/f; generously provided by T. Ishikawa and H. Herschman, UCLA) and with or without a Lyz2-Cre transgene (Lyz2^tm1(cre)Ifo^, Jackson Laboratory) were used to obtain femur samples for immunohistochemical detection of COX-2 and identification of osteoclasts by TRAP staining in serial sections. The Lyz2-Cre transgene expresses Cre recombinase in monocyte-derived cells and should therefore create a null allele of COX-2 in osteoclasts. Bone surface, TRAP-positive cells were detected in samples from mice of both genotypes. However, COX-2 expression was diminished in the apparent osteoclasts but not in the bone marrow leukocytes of the COX-2 f/f; Lyz2-Cre mice.
(TIF)

File S1 Tables S1–S23 summarize cell counting methods, immunohistochemistry cell counting results, inter-observer variation analysis of Day 7 specimens, and statistical analyses.
(DOCX)
