# Supplementary figures and images for: Immunohistochemical Localization of Key Arachidonic Acid Metabolism Enzymes during Fracture Healing in Mice
Source: PLoS One. 2014 Feb 7;9(2):e88423. doi: 10.1371/journal.pone.0088423 (PMC3917894; doi:10.1371/journal.pone.0088423)

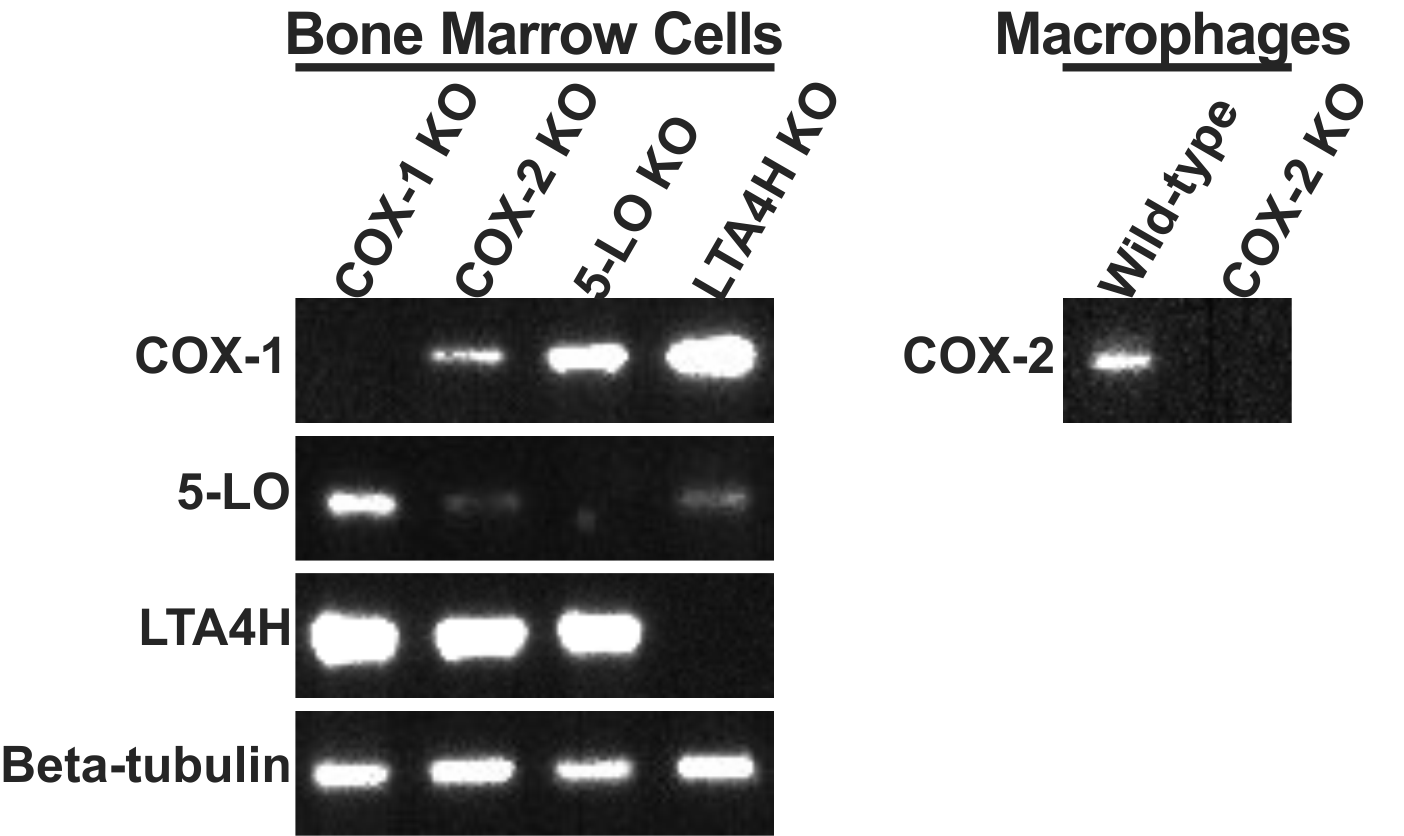

Supplement: Figure S1 — Antibody Specificity. The specificity of the antibodies used to detect COX-1, COX-2, 5-LO, and LTA4H was verified be using bone marrow derived cells and macrophages. Total protein extracts prepared from the bone marrow of COX-1, COX-2, 5-LO, and LTA4H knockout mice were analyzed for COX-1, 5-LO, and LTA4H expression by immunoblot analysis (Bone Marrow Cells). Expression of ß-tubulin was used as a control. The antibodies for COX-1, 5-LO, and LTA4H failed to detect any protein in their corresponding knockout bone marrow cell extracts but did detect proteins of the correct size (markers not shown) in the other bone marrow cell extracts. To detect COX-2 expression, bone marrow cells from wild-type and COX-2 knockout mice were cultured in DMEM with 10% FBS and 20% L929 conditioned media to promote macrophage development (Macrophages). The cultures were then induced to express COX-2 by treating with 100 ng/ml of LPS overnight followed by protein extract preparation and immunoblot analysis for COX-2 expression. The COX-2 antibody detected a protein of the correct size in the LPS-treated wild-type cells but failed to detect any protein in the COX-2 knockout cells. Antibodies were detected by chemiluminescence using appropriate horseradish peroxidase conjugated secondary antibodies and a Proteinsimple Fluorchem M imaging system. (TIF) [file pone.0088423.s001.tif]

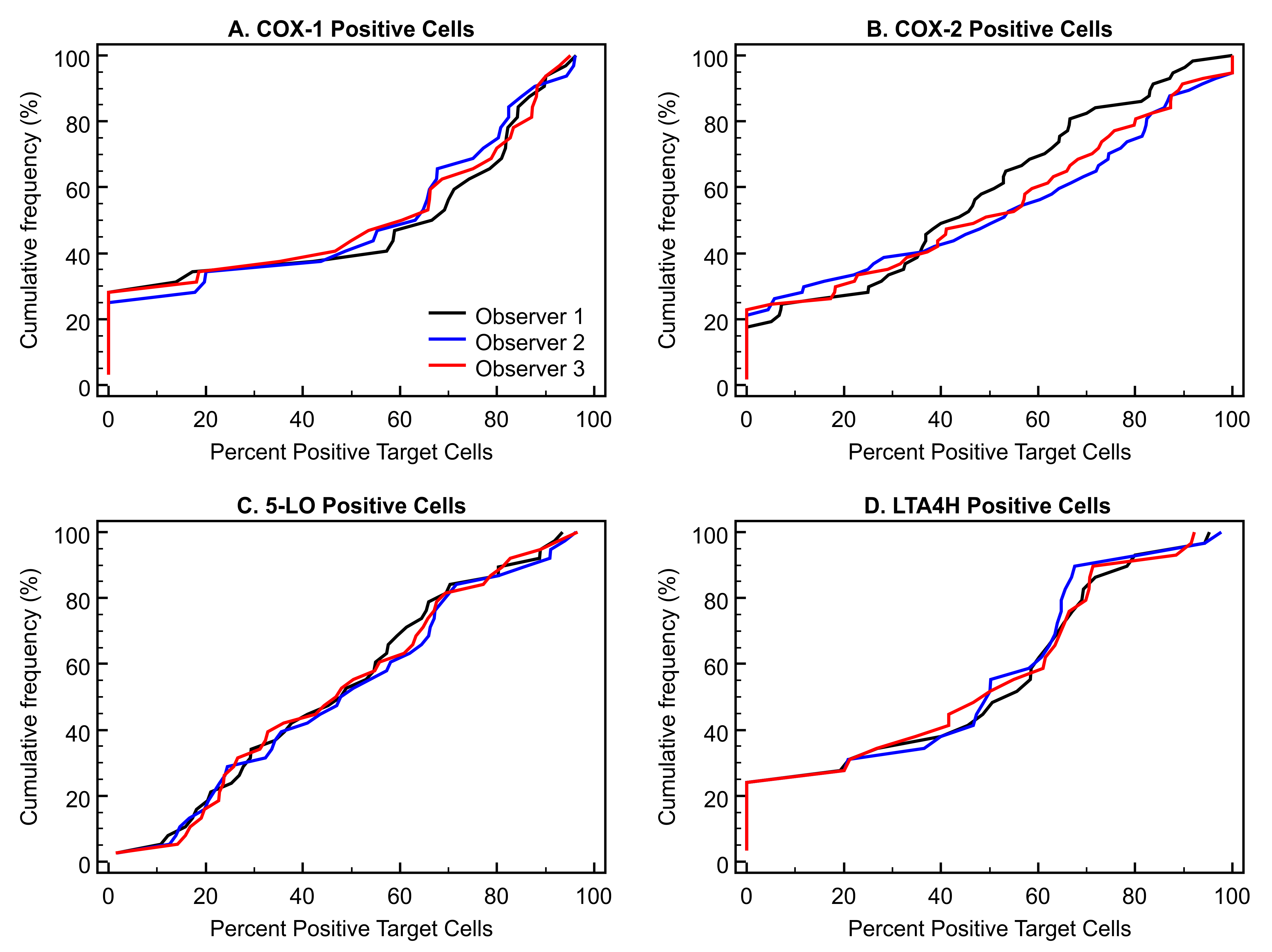

Supplement: Figure S2 — Inter-Observer Variation for Each Antibody. Consistency between observers for the percentage of antibody positive cells counted in each sample was determined using intraclass correlation coefficient tests. The cumulative frequency distribution for each observer (or rater) and each antibody (A: COX-1, B: COX-2, C: 5-LO, and D: LTA4H) are shown with maximum 100% positive target cells. (TIF) [file pone.0088423.s002.tif]

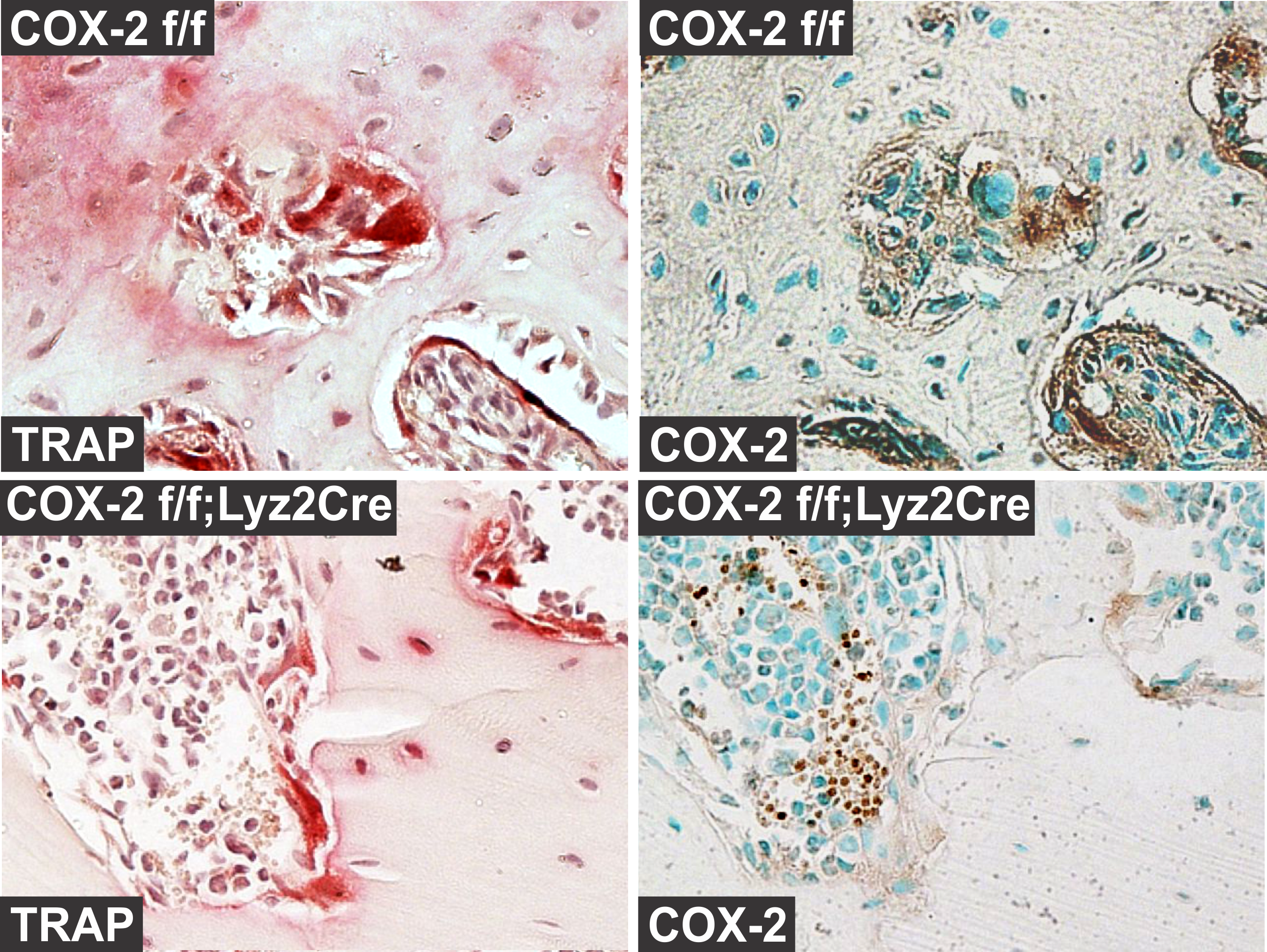

Supplement: Figure S3 — Expression of COX-2 in Osteoclasts. Mice homozygous for a floxed allele of COX-2 (COX-2 f/f; generously provided by T. Ishikawa and H. Herschman, UCLA) and with or without a Lyz2-Cre transgene (Lyz2tm1(cre)Ifo, Jackson Laboratory) were used to obtain femur samples for immunohistochemical detection of COX-2 and identification of osteoclasts by TRAP staining in serial sections. The Lyz2-Cre transgene expresses Cre recombinase in monocyte-derived cells and should therefore create a null allele of COX-2 in osteoclasts. Bone surface, TRAP-positive cells were detected in samples from mice of both genotypes. However, COX-2 expression was diminished in the apparent osteoclasts but not in the bone marrow leukocytes of the COX-2 f/f; Lyz2-Cre mice. (TIF) [file pone.0088423.s003.tif]
