# Supplementary material for: Immunohistochemical Localization of Key Arachidonic Acid Metabolism Enzymes during Fracture Healing in Mice
Source: PLoS One. 2014 Feb 7;9(2):e88423. doi: 10.1371/journal.pone.0088423 (PMC3917894; doi:10.1371/journal.pone.0088423)
Supplement: File S1 — Tables S1–S23 summarize cell counting methods, immunohistochemistry cell counting results, inter-observer variation analysis of Day 7 specimens, and statistical analyses. (DOCX) [file pone.0088423.s004.docx]

**The Tables listed below can be found in the following pages.**

**Table S1.** Summary of Cell Counting Methodology

**Table S2.** Intraclass Correlation Coefficients (ICC) and 95% Confidence Intervals (CI) for Observer-Related Variation in Cell Counts for Day 7 Specimens

**Tables S3-S13.** These tables summarize sample sizes, the mean number of enzyme positive cells counted based upon immunohistochemical detection, the mean number of cells of that type counted based upon morphological criteria, the mean percent enzyme positive cells, and associated standard deviation. The percentage of enzyme positive cells were compared for each enzyme and cell type over the time course of healing using ANOVA and post-hoc Holm-Sidak tests if the ANOVA indicated significance. The P values from those analyses are shown. Significant P values are bolded. (ND = None Detected; NA = Not Applicable)

**Tables S14-S23**. These tables summarize the percent cell counts obtained by 3 independent observers (examiners) for each antibody and cell type at day 7 after fracture. The data summarized below were used to assess interobserver variability.

**Table S1. Summary of Cell Counting Methodology**

| **Cell Type** | **No. of Images per Sample** | **Image Area or Length** | **Counting Areas per Image** | **No. of Counting Areas per Sample** | **Counting Area or Summed Length** | **Counting Method** |
| --- | --- | --- | --- | --- | --- | --- |
| Marrow Cells | 5 | 142804 um^2^ | 1 | 5 | 5625 um^2^ | The target cell type within the counting area was counted to obtain the total number of target cells within that area. Within the target cell type, the number of enzyme-positive cells within the counting area were counted. The percentage of enzyme-positive cells within each counting area was determined and averaged with all counting areas for that sample to obtain the mean percent of enzyme-positive target cells for that sample.  For the endosteal and periosteal cells, the number of enzyme-positive cells was divided by the length of entire endosteal or periosteal surface to obtain the number of enzyme-positive cells per unit length of bone surface for each sample.  Each sample represents the fracture callus from one mouse. Sample values for each target cell type were averaged within a time point for analysis and statistical comparisons. |
| Chondrocytes | 5 | 142804 um^2^ | 1 | 5 | 40000 um^2^ |  |
| Intramedullary Fracture Site | 5 | 142804 um^2^ | 1 | 5 | 5625 um^2^ |  |
| Muscle Interstitial Cells | 5 | 142804 um^2^ | 1 | 5 | 5625 um^2^ |  |
| Callus Leukocytes | 5 | 142804 um^2^ | 1 | 5 | 5625 um^2^ |  |
| Callus Periosteal Leukocytes | 5 | 142804 um^2^ | 1 | 5 | 5625 um^2^ |  |
| Periosteal Cells | 26-28 | 872.8 um | 1 | 26-28 | 23270-25060 um* |  |
| Endosteal Cells | 21-23 | 872.8 um | 1 | 21-23 | 19173-20999 um* |  |
| External Callus Osteoclasts | 5 | 142804 um^2^ | 1 | 5 | 40000 um^2^ |  |
| Internal Callus Osteoclasts | 5 | 142804 um^2^ | 1 | 5 | 40000 um^2^ |  |
| Fibroblasts | 5 | 142804 um^2^ | 1 | 5 | 5625 um^2^ |  |
| TRAP Positive Cells | 5 | 142804 um^2^ | 1 | 5 | 40000 um^2^ |  |

*The summed counting length of each sample is longer than the sum of image lengths due to the contour of bone surface.

**Table S2. Intraclass Correlation Coefficients (ICC) and 95% Confidence Intervals (CI) for Observer-Related Variation in Cell Counts for Day 7 Specimens**

| **Target Enzyme** | **No. Raters** | **No. Subjects** | **Single Measures ICC** | **Single Measures 95% CI** | **Average Measures ICC** | **Average Measures 95% CI** |
| --- | --- | --- | --- | --- | --- | --- |
| COX-1 | 3 | 32 | 0.95 | 0.91-0.97 | 0.98 | 0.97-0.99 |
| COX-2 | 3 | 57 | 0.87 | 0.81-0.92 | 0.95 | 0.93-0.97 |
| 5-LO | 3 | 38 | 0.99 | 0.98-0.99 | 1.00 | 0.99-1.00 |
| LTA4H | 3 | 29 | 0.96 | 0.93-0.98 | 0.99 | 0.97-0.99 |

| **Table S3. Marrow Cells** | | | | | | | | | | | | | |
| --- | --- | --- | --- | --- | --- | --- | --- | --- | --- | --- | --- | --- | --- |
| COX-1, ANOVA: P<0.001, Holm-Sidak method | | | | | | | | | | | | | |
| Time | sample  size | Mean (Positive Cell Number per 5625 um^2^) | Mean (Total Cell Number per 5625 um^2^) | Mean (Percent Positive) | Std.Dev. (Percent Positive) | P value | | | | | | | |
|  |  |  |  |  |  | 0 Hour | 6 Hours | Day 1 | Day 2 | Day 4 | Day 7 | Day 10 | Day 14 |
| 0 Hour | 6 | 40 | 107 | 37.9 | 2.4 | NA |  |  |  |  |  |  |  |
| 6 Hours | 6 | 51 | 112 | 45.7 | 7.1 | 0.404 | NA |  |  |  |  |  |  |
| Day 1 | 6 | 51 | 96 | 53.0 | 7.9 | **0.011** | 0.363 | NA |  |  |  |  |  |
| Day 2 | 6 | 51 | 96 | 52.6 | 5.4 | **0.013** | 0.356 | 0.925 | NA |  |  |  |  |
| Day 4 | 7 | 61 | 94 | 65.2 | 2.9 | **<0.001** | **<0.001** | **0.04** | **0.034** | NA |  |  |  |
| Day 7 | 6 | 78 | 111 | 70.3 | 15.6 | **<0.001** | **<0.001** | **0.002** | **0.002** | 0.516 | NA |  |  |
| Day 10 | 6 | 34 | 100 | 34.4 | 5.5 | 0.647 | 0.088 | **0.001** | **0.001** | **<0.001** | **<0.001** | NA |  |
| Day 14 | 7 | 23 | 84 | 27.3 | 2.5 | 0.087 | **<0.001** | **<0.001** | **<0.001** | **<0.001** | **<0.001** | 0.403 | NA |
| COX-2, ANOVA: P<0.001, Holm-Sidak method | | | | | | | | | | | | | |
| Time | sample  size | Mean (Positive Cell Number per 5625 um^2^) | Mean (Total Cell Number per 5625 um^2^) | Mean (Percent Positive) | Std.Dev. (Percent Positive | P value | | | | | | | |
|  |  |  |  |  |  | 0 Hour | 6 Hours | Day 1 | Day 2 | Day 4 | Day 7 | Day 10 | Day 14 |
| 0 Hour | 6 | 20 | 115 | 17.5 | 8.2 | NA |  |  |  |  |  |  |  |
| 6 Hours | 6 | 70 | 113 | 61.5 | 6.9 | **<0.001** | NA |  |  |  |  |  |  |
| Day 1 | 6 | 71 | 104 | 67.7 | 5.9 | **<0.001** | 0.724 | NA |  |  |  |  |  |
| Day 2 | 6 | 58 | 96 | 60.0 | 3.8 | **<0.001** | 0.749 | 0.58 | NA |  |  |  |  |
| Day 4 | 6 | 55 | 98 | 56.6 | 11.7 | **<0.001** | 0.871 | 0.19 | 0.912 | NA |  |  |  |
| Day 7 | 7 | 74 | 117 | 63.2 | 10.5 | **<0.001** | 0.9 | 0.834 | 0.843 | 0.686 | NA |  |  |
| Day 10 | 6 | 35 | 100 | 35.5 | 6.4 | **0.003** | **<0.001** | **<0.001** | **<0.001** | **<0.001** | **<0.001** | NA |  |
| Day 14 | 6 | 24 | 93 | 26.1 | 4.2 | 0.471 | **<0.001** | **<0.001** | **<0.001** | **<0.001** | **<0.001** | 0.38 | NA |
| 5-LO, ANOVA: P<0.001, Holm-Sidak method | | | | | | | | | | | | | |
| Time | sample  size | Mean (Positive Cell Number per 5625 um^2^) | Mean (Total Cell Number per 5625 um^2^) | Mean (Percent Positive) | Std.Dev. (Percent Positive | P value | | | | | | | |
|  |  |  |  |  |  | 0 Hour | 6 Hours | Day 1 | Day 2 | Day 4 | Day 7 | Day 10 | Day 14 |
| 0 Hour | 6 | 54 | 122 | 44.4 | 8.3 | NA |  |  |  |  |  |  |  |
| 6 Hours | 6 | 51 | 139 | 36.6 | 11.5 | 0.855 | NA |  |  |  |  |  |  |
| Day 1 | 6 | 49 | 107 | 45.7 | 11.0 | 0.838 | 0.769 | NA |  |  |  |  |  |
| Day 2 | 6 | 57 | 119 | 47.7 | 7.2 | 0.996 | 0.605 | 0.937 | NA |  |  |  |  |
| Day 4 | 6 | 80 | 107 | 74.1 | 13.3 | **<0.001** | **<0.001** | **<0.001** | **0.002** | NA |  |  |  |
| Day 7 | 6 | 96 | 124 | 77.0 | 17.0 | **<0.001** | **<0.001** | **<0.001** | **<0.001** | 0.983 | NA |  |  |
| Day 10 | 6 | 34 | 98 | 34.5 | 7.4 | 0.72 | 0.983 | 0.631 | 0.437 | **<0.001** | **<0.001** | NA |  |
| Day 14 | 6 | 24 | 77 | 31.3 | 5.2 | 0.42 | 0.972 | 0.323 | 0.172 | **<0.001** | **<0.001** | 0.991 | NA |
| LTA4H, ANOVA: P<0.001, Holm-Sidak method | | | | | | | | | | | | | |
| Time | sample  size | Mean (Positive Cell Number per 5625 um^2^) | Mean (Total Cell Number per 5625 um^2^) | Mean (Percent Positive) | Std.Dev. (Percent Positive) | P value | | | | | | | |
|  |  |  |  |  |  | 0 Hour | 6 Hours | Day 1 | Day 2 | Day 4 | Day 7 | Day 10 | Day 14 |
| 0 Hour | 6 | 37 | 99 | 36.9 | 2.0 | NA |  |  |  |  |  |  |  |
| 6 Hours | 6 | 44 | 101 | 43.5 | 7.1 | 0.26 | NA |  |  |  |  |  |  |
| Day 1 | 6 | 46 | 92 | 49.9 | 7.9 | **0.005** | 0.43 | NA |  |  |  |  |  |
| Day 2 | 6 | 45 | 89 | 50.5 | 5.4 | **0.007** | 0.365 | 0.926 | NA |  |  |  |  |
| Day 4 | 7 | 59 | 90 | 65.2 | 2.9 | **<0.001** | **<0.001** | **0.044** | **0.037** | NA |  |  |  |
| Day 7 | 6 | 74 | 111 | 66.6 | 15.6 | **<0.001** | **<0.001** | **0.003** | **0.002** | 0.525 | NA |  |  |
| Day 10 | 6 | 40 | 118 | 34.2 | 5.5 | 0.818 | 0.094 | **0.001** | **0.002** | **<0.001** | **<0.001** | NA |  |
| Day 14 | 6 | 25 | 96 | 26.6 | 2.7 | 0.206 | **0.001** | **<0.001** | **<0.001** | **<0.001** | **<0.001** | 0.396 | NA |

| **Table S4. Chondrocytes** | | | | | | | | | |
| --- | --- | --- | --- | --- | --- | --- | --- | --- | --- |
| COX-1, ANOVA: P<0.001, Holm-Sidak method | | | | | | | | | |
| Time | sample  size | Mean  (Positive Cell Number per 40000 um^2^) | Mean  (Total Cell Number per 40000 um^2^) | Mean (Percent Positive) | Std.Dev. (Percent Positive | P value | | | |
|  |  |  |  |  |  | Day 4 | Day 7 | Day 10 | Day 14 |
| Day 4 | 6 | 65 | 73 | 88.7 | 10.8 | NA |  |  |  |
| Day 7 | 7 | 61 | 82 | 74.0 | 15.0 | 0.082 | NA |  |  |
| Day 10 | 6 | 28 | 48 | 58.0 | 16.3 | **0.006** | 0.173 | NA |  |
| Day 14 | 6 | 14 | 33 | 42.1 | 15.2 | **<0.001** | **0.004** | 0.137 | NA |
| COX-2, ANOVA: P<0.001, Holm-Sidak method | | | | | | | | | |
| Time | sample  size | Mean  (Positive Cell Number per 40000 um^2^) | Mean  (Total Cell Number per 40000 um^2^) | Mean (Percent Positive) | Std.Dev. (Percent Positive | P value | | | |
|  |  |  |  |  |  | Day 4 | Day 7 | Day 10 | Day 14 |
| Day 4 | 5 | 86 | 98 | 88.4 | 5.1 | NA |  |  |  |
| Day 7 | 8 | 78 | 90 | 87.1 | 8.6 | 0.797 | NA |  |  |
| Day 10 | 6 | 26 | 40 | 64.4 | 4.8 | **<0.001** | **<0.001** | NA |  |
| Day 14 | 6 | 10 | 33 | 32.0 | 13.3 | **<0.001** | **<0.001** | **<0.001** | NA |
| 5-LO, ANOVA: P=0.007, Holm-Sidak method | | | | | | | | | |
| Time | sample  size | Mean  (Positive Cell Number per 40000 um^2^) | Mean  (Total Cell Number per 40000 um^2^) | Mean (Percent Positive) | Std.Dev. (Percent Positive | P value | | | |
|  |  |  |  |  |  | Day 4 | Day 7 | Day 10 | Day 14 |
| Day 4 | 6 | 59 | 75 | 78.6 | 6.7 | NA |  |  |  |
| Day 7 | 8 | 45 | 94 | 47.4 | 26.2 | 0.054 | NA |  |  |
| Day 10 | 6 | 18 | 51 | 34.5 | 25.4 | **0.011** | 0.627 | NA |  |
| Day 14 | 6 | 12 | 31 | 38.3 | 19.8 | **0.019** | 0.691 | 0.763 | NA |
| LTA4H, ANOVA: P=0.001, Holm-Sidak method | | | | | | | | | |
| Time | sample  size | Mean  (Positive Cell Number per 40000 um^2^) | Mean  (Total Cell Number per 40000 um^2^) | Mean (Percent Positive) | Std.Dev. (Percent Positive | P value | | | |
|  |  |  |  |  |  | Day 4 | Day 7 | Day 10 | Day 14 |
| Day 4 | 6 | 35 | 58 | 59.6 | 8.5 | NA |  |  |  |
| Day 7 | 6 | 44 | 70 | 62.5 | 2.8 | 0.617 | NA |  |  |
| Day 10 | 7 | 22 | 58 | 38.8 | 13.9 | **0.005** | **0.002** | NA |  |
| Day 14 | 6 | 25 | 46 | 54.1 | 9.3 | 0.563 | 0.39 | **0.04** | NA |

| **Table S5. Intramedullary Fracture Site** | | | | | | | | | | | | |
| --- | --- | --- | --- | --- | --- | --- | --- | --- | --- | --- | --- | --- |
| COX-1, ANOVA: P<0.001, Holm-Sidak method | | | | | | | | | | | | |
| Time | sample  size | Mean (Positive Cell Number per 5625 um^2^) | Mean (Total Cell Number per 5625 um^2^) | Mean (Percent Positive) | Std.Dev. (Percent Positive | P value | | | | | | |
|  |  |  |  |  |  | 6 Hours | Day 1 | Day 2 | Day 4 | Day 7 | Day 10 | Day 14 |
| 6 Hours | 6 | 50 | 75 | 66.0 | 9.8 | NA |  |  |  |  |  |  |
| Day 1 | 6 | 13 | 28 | 45.1 | 12.1 | **0.001** | N/A |  |  |  |  |  |
| Day 2 | 6 | 7 | 18 | 39.3 | 16.0 | **<0.001** | 0.764 | NA |  |  |  |  |
| Day 4 | 6 | 4 | 32 | 12.2 | 4.6 | **<0.001** | **<0.001** | **<0.001** | NA |  |  |  |
| Day 7 | 6 | 1 | 21 | 3.0 | 7.3 | **<0.001** | **<0.001** | **<0.001** | 0.433 | NA |  |  |
| Day 10 | 6 | 0.03 | 9 | 0.4 | 0.9 | **<0.001** | **<0.001** | **<0.001** | 0.174 | 0.839 | NA |  |
| Day 14 | 6 | 1 | 20 | 6.0 | 1.6 | **<0.001** | **<0.001** | **<0.001** | 0.771 | 0.902 | 0.695 | NA |
| COX-2, ANOVA: P<0.001, Holm-Sidak method | | | | | | | | | | | | |
| Time | sample  size | Mean (Positive Cell Number per 5625 um^2^) | Mean (Total Cell Number per 5625 um^2^) | Mean (Percent Positive) | Std.Dev. (Percent Positive | P value | | | | | | |
|  |  |  |  |  |  | 6 Hours | Day 1 | Day 2 | Day 4 | Day 7 | Day 10 | Day 14 |
| 6 Hours | 6 | 16 | 72 | 22.4 | 7.3 | NA |  |  |  |  |  |  |
| Day 1 | 6 | 11 | 35 | 31.7 | 9.2 | 0.052 | NA |  |  |  |  |  |
| Day 2 | 6 | 5 | 23 | 24.0 | 4.5 | 0.859 | 0.152 | NA |  |  |  |  |
| Day 4 | 6 | 4 | 36 | 12.1 | 3.7 | **0.024** | **<0.001** | **0.007** | NA |  |  |  |
| Day 7 | 6 | 0.4 | 23 | 1.6 | 2.3 | **<0.001** | **<0.001** | **<0.001** | **0.022** | NA |  |  |
| Day 10 | 6 | 0 | ND | 0.0 | 0.0 | **<0.001** | **<0.001** | **<0.001** | **0.006** | 0.941 | NA |  |
| Day 14 | 6 | 0 | ND | 0.0 | 0.0 | **<0.001** | **<0.001** | **<0.001** | **0.007** | 0.977 | 1 | NA |
| 5-LO, ANOVA: P<0.001, Holm-Sidak method | | | | | | | | | | | | |
| Time | sample  size | Mean (Positive Cell Number per 5625 um^2^) | Mean (Total Cell Number per 5625 um^2^) | Mean (Percent Positive) | Std.Dev. (Percent Positive | P value | | | | | | |
|  |  |  |  |  |  | 6 Hours | Day 1 | Day 2 | Day 4 | Day 7 | Day 10 | Day 14 |
| 6 Hours | 6 | 25 | 77 | 32.3 | 12.2 | NA |  |  |  |  |  |  |
| Day 1 | 6 | 11 | 34 | 32.3 | 10.9 | 1 | NA |  |  |  |  |  |
| Day 2 | 6 | 9 | 25 | 34.1 | 22.4 | 0.987 | 0.997 | NA |  |  |  |  |
| Day 4 | 6 | 6 | 33 | 17.0 | 5.2 | 0.15 | 0.141 | 0.092 | NA |  |  |  |
| Day 7 | 6 | 4 | 25 | 14.7 | 7.0 | 0.086 | 0.083 | **0.044** | 0.998 | NA |  |  |
| Day 10 | 8 | 0 | ND | 0.0 | 0.0 | **<0.001** | **<0.001** | **<0.001** | 0.072 | 0.139 | NA |  |
| Day 14 | 6 | 0 | ND | 0.0 | 0.0 | **<0.001** | **<0.001** | **<0.001** | 0.091 | 0.157 | 1 | NA |
| LTA4H, ANOVA: P<0.001, Holm-Sidak method | | | | | | | | | | | | |
| Time | sample  size | Mean (Positive Cell Number per 5625 um^2^) | Mean (Total Cell Number per 5625 um^2^) | Mean (Percent Positive) | Std.Dev. (Percent Positive | P value | | | | | | |
|  |  |  |  |  |  | 6 Hours | Day 1 | Day 2 | Day 4 | Day 7 | Day 10 | Day 14 |
| 6 Hours | 6 | 42 | 80 | 52.5 | 7.5 | NA |  |  |  |  |  |  |
| Day 1 | 6 | 12 | 36 | 33.9 | 13.7 | **0.003** | NA |  |  |  |  |  |
| Day 2 | 6 | 4 | 19 | 21.5 | 9.9 | **<0.001** | 0.062 | NA |  |  |  |  |
| Day 4 | 6 | 5 | 35 | 15.2 | 12.8 | **<0.001** | **0.003** | 0.617 | NA |  |  |  |
| Day 7 | 6 | 0 | ND | 0.0 | 0.0 | **<0.001** | **<0.001** | **<0.001** | **0.019** | NA |  |  |
| Day 10 | 6 | 0 | ND | 0.0 | 0.0 | **<0.001** | **<0.001** | **<0.001** | **0.017** | 1 | NA |  |
| Day 14 | 6 | 0 | ND | 0.0 | 0.0 | **<0.001** | **<0.001** | **<0.001** | **0.015** | 1 | 1 | NA |

| **Table S6. Muscle Interstitial Cells** | | | | | | | | | | | | | |
| --- | --- | --- | --- | --- | --- | --- | --- | --- | --- | --- | --- | --- | --- |
| COX-1, ANOVA: P<0.001, Holm-Sidak method | | | | | | | | | | | | | |
| Time | sample  size | Mean (Positive Cell Number per 5625 um^2^) | Mean (Total Cell Number per 5625 um^2^) | Mean (Percent Positive) | Std.Dev. (Percent Positive | P value | | | | | | | |
|  |  |  |  |  |  | 0 Hour | 6 Hours | Day 1 | Day 2 | Day 4 | Day 7 | Day 10 | Day 14 |
| 0 Hour | 6 | 0.1 | 0.8 | 6.8 | 16.7 | NA |  |  |  |  |  |  |  |
| 6 Hours | 6 | 4 | 7 | 59.4 | 30.7 | **<0.001** | NA |  |  |  |  |  |  |
| Day 1 | 6 | 9 | 12 | 80.5 | 10.2 | **<0.001** | 0.345 | N/A |  |  |  |  |  |
| Day 2 | 6 | 8 | 18 | 45.7 | 15.6 | **0.004** | 0.83 | **0.012** | NA |  |  |  |  |
| Day 4 | 8 | 1 | 7 | 15.7 | 18.1 | 0.941 | **<0.001** | **<0.001** | **0.025** | NA |  |  |  |
| Day 7 | 6 | 0.1 | 4 | 3.4 | 8.2 | 0.922 | **<0.001** | **<0.001** | **0.001** | 0.832 | NA |  |  |
| Day 10 | 6 | 0.2 | 2 | 12.1 | 16.2 | 0.972 | **<0.001** | **<0.001** | **0.017** | 0.97 | 0.94 | NA |  |
| Day 14 | 6 | 0 | ND | 0.0 | 0.0 | 0.963 | **<0.001** | **<0.001** | **<0.001** | 0.647 | 0.731 | 0.863 | NA |
| COX-2, ANOVA: P<0.001, Holm-Sidak method | | | | | | | | | | | | | |
| Time | sample  size | Mean (Positive Cell Number per 5625 um^2^) | Mean (Total Cell Number per 5625 um^2^) | Mean (Percent Positive) | Std.Dev. (Percent Positive | P value | | | | | | | |
|  |  |  |  |  |  | 0 Hour | 6 Hours | Day 1 | Day 2 | Day 4 | Day 7 | Day 10 | Day 14 |
| 0 Hour | 6 | 0 | ND | 0.0 | 0.0 | NA |  |  |  |  |  |  |  |
| 6 Hours | 6 | 4 | 7 | 61.2 | 17.5 | **<0.001** | NA |  |  |  |  |  |  |
| Day 1 | 6 | 5 | 16 | 32.8 | 17.4 | **<0.001** | **0.001** | NA |  |  |  |  |  |
| Day 2 | 6 | 3 | 18 | 19.0 | 15.5 | 0.075 | **<0.001** | 0.36 | NA |  |  |  |  |
| Day 4 | 6 | 1 | 11 | 12.5 | 10.1 | 0.475 | **<0.001** | **0.047** | 0.929 | NA |  |  |  |
| Day 7 | 7 | 0.3 | 5 | 4.6 | 6.6 | 0.913 | **<0.001** | **<0.001** | 0.274 | 0.844 | NA |  |  |
| Day 10 | 6 | 0 | ND | 0.0 | 0.0 | 1 | **<0.001** | **<0.001** | 0.071 | 0.41 | 0.953 | NA |  |
| Day 14 | 6 | 0 | ND | 0.0 | 0.0 | 1 | **<0.001** | **<0.001** | 0.066 | 0.444 | 0.974 | 1 | NA |
| 5-LO, ANOVA: P<0.001, Holm-Sidak method | | | | | | | | | | | | | |
| Time | sample  size | Mean (Positive Cell Number per 5625 um^2^) | Mean (Total Cell Number per 5625 um^2^) | Mean (Percent Positive) | Std.Dev. (Percent Positive | P value | | | | | | | |
|  |  |  |  |  |  | 0 Hour | 6 Hours | Day 1 | Day 2 | Day 4 | Day 7 | Day 10 | Day 14 |
| 0 Hour | 6 | 0.03 | 0.5 | 6.1 | 11.2 | NA |  |  |  |  |  |  |  |
| 6 Hours | 6 | 3 | 6 | 41.9 | 39.5 | 0.052 | NA |  |  |  |  |  |  |
| Day 1 | 6 | 6 | 14 | 46.7 | 24.7 | **0.022** | 1 | NA |  |  |  |  |  |
| Day 2 | 6 | 5 | 13 | 41.1 | 25.1 | 0.058 | 0.948 | 1 | NA |  |  |  |  |
| Day 4 | 6 | 6 | 11 | 57.1 | 13.7 | **0.002** | 0.929 | 0.991 | 0.92 | NA |  |  |  |
| Day 7 | 6 | 3 | 6 | 44.2 | 12.6 | **0.036** | 0.999 | 1 | 1 | 0.971 | NA |  |  |
| Day 10 | 8 | 0.1 | 3 | 2.8 | 7.9 | 1 | **0.018** | **0.006** | **0.021** | **<0.001** | **0.01** | NA |  |
| Day 14 | 6 | 0.03 | 0.7 | 4.2 | 5.8 | 0.998 | **0.038** | **0.016** | **0.042** | **0.001** | **0.024** | 0.99 | NA |
| LTA4H, ANOVA: P<0.001, Holm-Sidak method | | | | | | | | | | | | | |
| Time | sample  size | Mean (Positive Cell Number per 5625 um^2^) | Mean (Total Cell Number per 5625 um^2^) | Mean (Percent Positive) | Std.Dev. (Percent Positive | P value | | | | | | | |
|  |  |  |  |  |  | 0 Hour | 6 Hours | Day 1 | Day 2 | Day 4 | Day 7 | Day 10 | Day 14 |
| 0 Hour | 6 | 0 | ND | 0.0 | 0.0 | NA |  |  |  |  |  |  |  |
| 6 Hours | 6 | 7 | 14 | 50.9 | 27.6 | **<0.001** | NA |  |  |  |  |  |  |
| Day 1 | 6 | 8 | 13 | 60.5 | 26.4 | **<0.001** | 0.936 | NA |  |  |  |  |  |
| Day 2 | 6 | 7 | 13 | 55.9 | 16.9 | **<0.001** | 0.99 | 0.983 | NA |  |  |  |  |
| Day 4 | 7 | 1 | 7 | 17.2 | 11.5 | 0.447 | **0.014** | **<0.001** | **0.003** | NA |  |  |  |
| Day 7 | 6 | 0.9 | 4 | 23.7 | 15.4 | 0.201 | 0.098 | **0.008** | **0.028** | 0.983 | NA |  |  |
| Day 10 | 6 | 0.8 | 4 | 21.2 | 12.3 | 0.315 | 0.054 | **0.004** | **0.014** | 0.964 | 0.96 | NA |  |
| Day 14 | 6 | 0 | ND | 0.0 | 0.0 | 1 | **<0.001** | **<0.001** | **<0.001** | 0.486 | 0.216 | 0.291 | NA |

| **Table S7. Callus Leukocytes** | | | | | | | | | |
| --- | --- | --- | --- | --- | --- | --- | --- | --- | --- |
| COX-1, ANOVA: P<0.001, Holm-Sidak method | | | | | | | | | |
| Time | sample  size | Mean (Positive Cell Number per 5625 um^2^) | Mean (Total Cell Number per 5625 um^2^) | Mean (Percent Positive) | Std.Dev. (Percent Positive | P value | | | |
|  |  |  |  |  |  | Day 4 | Day 7 | Day 10 | Day 14 |
| Day 4 | 3 | 3 | 19 | 15.2 | 11.2 | NA |  |  |  |
| Day 7 | 4 | 7 | 12 | 57.3 | 22.1 | **0.002** | NA |  |  |
| Day 10 | 5 | 9 | 20 | 45.0 | 3.5 | **0.013** | 0.275 | NA |  |
| Day 14 | 6 | 5 | 44 | 11.4 | 7.1 | 0.664 | **<0.001** | **0.002** | NA |
| COX-2, ANOVA: P=0.485 | | | | | |  |  |  |  |
| Time | sample  size | Mean (Positive Cell Number per 5625 um^2^) | Mean (Total Cell Number per 5625 um^2^) | Mean (Percent Positive) | Std.Dev. (Percent Positive |  | | | |
| Day 4 | 2 | 6 | 21 | 31.4 | 4.0 |  |  |  |  |
| Day 7 | 2 | 5 | 12 | 40.6 | 22.1 |  |  |  |  |
| Day 10 | 3 | 11 | 26 | 43.9 | 4.5 |  |  |  |  |
| Day 14 | 6 | 19 | 56 | 33.9 | 9.4 |  |  |  |  |
| 5-LO, ANOVA: P=0.478 | | | | | |  |  |  |  |
| Time | sample  size | Mean (Positive Cell Number per 5625 um^2^) | Mean (Total Cell Number per 5625 um^2^) | Mean (Percent Positive) | Std.Dev. (Percent Positive |  | | | |
| Day 4 | 3 | 5 | 18 | 28.9 | 3.8 |  |  |  |  |
| Day 7 | 3 | 5 | 13 | 34.8 | 12.5 |  |  |  |  |
| Day 10 | 5 | 8 | 22 | 38.7 | 9.4 |  |  |  |  |
| Day 14 | 6 | 16 | 43 | 36.4 | 6.7 |  |  |  |  |
| LTA4H, ANOVA: P=0.067 | | | | | |  |  |  |  |
| Time | sample  size | Mean (Positive Cell Number per 5625 um^2^) | Mean (Total Cell Number per 5625 um^2^) | Mean (Percent Positive) | Std.Dev. (Percent Positive |  | | | |
| Day 4 | 7 | 7 | 18 | 40.6 | 33.2 |  |  |  |  |
| Day 7 | 3 | 5 | 11 | 43.5 | 6.1 |  |  |  |  |
| Day 10 | 4 | 18 | 24 | 76.0 | 9.8 |  |  |  |  |
| Day 14 | 6 | 12 | 32 | 38.8 | 10.4 |  |  |  |  |

| **Table S8. Callus Periosteal Leukocytes** | | | | | | | | | | | | |
| --- | --- | --- | --- | --- | --- | --- | --- | --- | --- | --- | --- | --- |
| COX-1, ANOVA: P<0.001, Holm-Sidak method | | | | | | | | | | | | |
| Time | sample  size | Mean (Positive Cell Number per 5625 um^2^) | Mean (Total Cell Number per 5625 um^2^) | Mean (Percent Positive) | Std.Dev. (Percent Positive | P value | | | | | | |
|  |  |  |  |  |  | 6 Hours | Day 1 | Day 2 | Day 4 | Day 7 | Day 10 | Day 14 |
| 6 Hours | 6 | 24 | 28 | 84.7 | 11.5 | NA |  |  |  |  |  |  |
| Day 1 | 6 | 19 | 21 | 89.4 | 13.1 | 0.907 | N/A |  |  |  |  |  |
| Day 2 | 6 | 14 | 22 | 64.9 | 19.7 | 0.157 | **0.044** | NA |  |  |  |  |
| Day 4 | 8 | 10 | 19 | 54.2 | 11.7 | **0.003** | **<0.001** | 0.725 | NA |  |  |  |
| Day 7 | 6 | 32 | 38 | 82.4 | 14.8 | 0.772 | 0.904 | 0.267 | **0.007** | NA |  |  |
| Day 10 | 6 | 50 | 52 | 94.9 | 6.7 | 0.784 | 0.929 | **0.007** | **<0.001** | 0.672 | NA |  |
| Day 14 | 6 | 20 | 33 | 62.3 | 14.0 | 0.084 | **0.019** | 0.933 | 0.85 | 0.155 | **0.003** | NA |
| COX-2, ANOVA: P<0.001, Holm-Sidak method | | | | | | | | | | | | |
| Time | sample  size | Mean (Positive Cell Number per 5625 um^2^) | Mean (Total Cell Number per 5625 um^2^) | Mean (Percent Positive) | Std.Dev. (Percent Positive | P value | | | | | | |
|  |  |  |  |  |  | 6 Hours | Day 1 | Day 2 | Day 4 | Day 7 | Day 10 | Day 14 |
| 6 Hours | 6 | 27 | 31 | 88.1 | 6.9 | NA |  |  |  |  |  |  |
| Day 1 | 6 | 11 | 22 | 50.2 | 8.7 | **<0.001** | NA |  |  |  |  |  |
| Day 2 | 6 | 6 | 24 | 25.3 | 10.7 | **<0.001** | **0.014** | NA |  |  |  |  |
| Day 4 | 8 | 8 | 21 | 39.9 | 15.6 | **<0.001** | 0.427 | 0.198 | NA |  |  |  |
| Day 7 | 6 | 23 | 37 | 63.9 | 17.1 | **0.016** | 0.281 | **<0.001** | **0.011** | NA |  |  |
| Day 10 | 6 | 31 | 43 | 72.5 | 13.2 | 0.223 | **0.031** | **<0.001** | **<0.001** | 0.412 | NA |  |
| Day 14 | 6 | 10 | 34 | 30.4 | 9.4 | **<0.001** | 0.067 | 0.481 | 0.419 | **<0.001** | **<0.001** | NA |
| LTA4H, ANOVA: P<0.001, Holm-Sidak method | | | | | | | | | | | | |
| Time | sample  size | Mean (Positive Cell Number per 5625 um^2^) | Mean (Total Cell Number per 5625 um^2^) | Mean (Percent Positive) | Std.Dev. (Percent Positive | P value | | | | | | |
|  |  |  |  |  |  | 6 Hours | Day 1 | Day 2 | Day 4 | Day 7 | Day 10 | Day 14 |
| 6 Hours | 6 | 29 | 33 | 88.7 | 13.0 | NA |  |  |  |  |  |  |
| Day 1 | 6 | 16 | 19 | 83.6 | 13.6 | 0.963 | NA |  |  |  |  |  |
| Day 2 | 6 | 20 | 25 | 79.4 | 11.2 | 0.873 | 0.949 | NA |  |  |  |  |
| Day 4 | 7 | 8 | 20 | 38.2 | 16.5 | **<0.001** | **<0.001** | **<0.001** | NA |  |  |  |
| Day 7 | 6 | 29 | 43 | 68.1 | 18.1 | 0.237 | 0.527 | 0.783 | **0.015** | NA |  |  |
| Day 10 | 6 | 53 | 62 | 86.4 | 5.6 | 0.795 | 0.937 | 0.937 | **<0.001** | 0.365 | NA |  |
| Day 14 | 6 | 21 | 38 | 54.4 | 21.1 | **0.006** | **0.024** | **0.08** | 0.457 | 0.65 | **0.011** | NA |
| 5-LO, ANOVA: P=0.056 | | | | | |  |  |  |  |  |  |  |
| Time | sample  size | Mean (Positive Cell Number per 5625 um^2^) | Mean (Total Cell Number per 5625 um^2^) | Mean (Percent Positive) | Std.Dev. (Percent Positive |  | | | | | | |
| 6 Hours | 6 | 18 | 33 | 56.0 | 26.9 |  |  |  |  |  |  |  |
| Day 1 | 6 | 12 | 24 | 50.8 | 19.4 |  |  |  |  |  |  |  |
| Day 2 | 6 | 9 | 19 | 45.2 | 23.2 |  |  |  |  |  |  |  |
| Day 4 | 6 | 9 | 18 | 50.3 | 9.1 |  |  |  |  |  |  |  |
| Day 7 | 6 | 23 | 33 | 70.0 | 13.8 |  |  |  |  |  |  |  |
| Day 10 | 6 | 43 | 57 | 75.3 | 15.9 |  |  |  |  |  |  |  |
| Day 14 | 6 | 18 | 30 | 62.2 | 9.4 |  |  |  |  |  |  |  |

| **Table S9. Periosteal Cells** | | | | | | | | | | | | | | | | | | | | | |  |  |
| --- | --- | --- | --- | --- | --- | --- | --- | --- | --- | --- | --- | --- | --- | --- | --- | --- | --- | --- | --- | --- | --- | --- | --- |
| COX-1, ANOVA: P<0.001, Holm-Sidak method | | | | | | | | | | | | | | | | | | | | | |  |  |
| Time | sample  size | Mean (Positive Cell Number per Specimen) | | | Mean (Total Cell Number per Specimen) | | | Mean (percent Positive) | Std.Dev. (Percent Positive | P value | | | | | | | | | | | |  |  |
|  |  |  | | |  | | |  |  | 0 Hour | 6 Hours | Day 1 | Day 2 | | Day 4 | | Day 7 | Day 10 | | Day 14 | |  |  |
| 0 Hour | 6 | 0 | | | ND | | | 0.0 | 0.0 | NA |  |  |  | |  | |  |  | |  | |  |  |
| 6 Hours | 6 | 233 | | | 1149 | | | 20.3 | 17.1 | **<0.001** | NA |  |  | |  | |  |  | |  | |  |  |
| Day 1 | 8 | 0 | | | ND | | | 0.0 | 0.0 | 1 | **<0.001** | N/A |  | |  | |  |  | |  | |  |  |
| Day 2 | 7 | 17 | | | 1036 | | | 1.7 | 4.4 | 1 | **<0.001** | 1 | NA | |  | |  |  | |  | |  |  |
| Day 4 | 8 | 0 | | | ND | | | 0.0 | 0.0 | 1 | **<0.001** | 1 | 1 | | NA | |  |  | |  | |  |  |
| Day 7 | 6 | 0 | | | ND | | | 0.0 | 0.0 | 1 | **<0.001** | 1 | 1 | | 1 | | NA |  | |  | |  |  |
| Day 10 | 6 | 0 | | | ND | | | 0.0 | 0.0 | 1 | **<0.001** | 1 | 1 | | 1 | | 1 | NA | |  | |  |  |
| Day 14 | 6 | 0 | | | ND | | | 0.0 | 0.0 | 1 | **<0.001** | 1 | 1 | | 1 | | 1 | 1 | | NA | |  |  |
| COX-2, ANOVA: P=0.002, Holm-Sidak method | | | | | | | | | | | | | | | | | | | | | |  |  |
| Time | sample  size | | Mean (Positive Cell Number per Specimen) | | | Mean (Total Cell Number per Specimen) | | Mean (percent Positive) | Std.Dev. (Percent Positive | P value | | | | | | | | | | | |  |  |
|  |  | |  | | |  | |  |  | 0 Hour | 6 Hours | Day 1 | Day 2 | | Day 4 | | Day 7 | Day 10 | | Day 14 | |  |  |
| 0 Hour | 6 | | 38 | | | 950 | | 4.0 | 3.2 | NA |  |  |  | |  | |  |  | |  | |  |  |
| 6 Hours | 7 | | 271 | | | 1144 | | 23.7 | 25.4 | 0.216 | NA |  |  | |  | |  |  | |  | |  |  |
| Day 1 | 6 | | 345 | | | 1244 | | 27.8 | 25.7 | 0.096 | 1 | NA |  | |  | |  |  | |  | |  |  |
| Day 2 | 6 | | 15 | | | 1116 | | 1.3 | 2.1 | 1 | 0.109 | **0.046** | NA | |  | |  |  | |  | |  |  |
| Day 4 | 6 | | 2 | | | 1416 | | 0.2 | 0.4 | 1 | 0.088 | **0.033** | 1 | | NA | |  |  | |  | |  |  |
| Day 7 | 6 | | 11 | | | 1458 | | 0.7 | 1.8 | 1 | 0.098 | **0.039** | 1 | | 0.997 | | NA |  | |  | |  |  |
| Day 10 | 5 | | 31 | | | 1447 | | 2.2 | 1.1 | 1 | 0.182 | 0.085 | 1 | | 1 | | 1 | NA | |  | |  |  |
| Day 14 | 6 | | 58 | | | 1430 | | 4.1 | 2.6 | 0.993 | 0.275 | 0.127 | 1 | | 1 | | 1 | 1 | | NA | |  |  |
| 5-LO, ANOVA: P=0.014, Holm-Sidak method | | | | | | | | | | | | | | | | | | | | | |  |  |
| Time | sample  size | | | Mean (Positive Cell Number per Specimen) | | | Mean (Total Cell Number per Specimen) | Mean (percent Positive) | Std.Dev. (Percent Positive | P value | | | | | | | | | | | |  |  |
|  |  | | |  | | |  |  |  | 0 Hour | 6 Hours | Day 1 | Day 2 | | Day 4 | | Day 7 | Day 10 | | Day 14 | |  |  |
| 0 Hour | 6 | | | 0 | | | ND | 0.0 | 0.0 | NA |  |  |  | |  | |  |  | |  | |  |  |
| 6 Hours | 6 | | | 295 | | | 1240 | 23.8 | 37.0 | 0.444 | NA |  |  | |  | |  |  | |  | |  |  |
| Day 1 | 6 | | | 400 | | | 1252 | 32.0 | 37.9 | 0.132 | 1 | NA |  | |  | |  |  | |  | |  |  |
| Day 2 | 6 | | | 0 | | | ND | 0.0 | 0.0 | 1 | 0.532 | 0.137 | NA | |  | |  |  | |  | |  |  |
| Day 4 | 6 | | | 0 | | | ND | 0.0 | 0.0 | 1 | 0.463 | 0.114 | 1 | | NA | |  |  | |  | |  |  |
| Day 7 | 6 | | | 0 | | | ND | 0.0 | 0.0 | 1 | 0.499 | 0.128 | 1 | | 1 | | NA |  | |  | |  |  |
| Day 10 | 6 | | | 0 | | | ND | 0.0 | 0.0 | 1 | 0.516 | 0.123 | 1 | | 1 | | 1 | NA | |  | |  |  |
| Day 14 | 6 | | | 0 | | | ND | 0.0 | 0.0 | 1 | 0.481 | 0.118 | 1 | | 1 | | 1 | 1 | | NA | |  |  |
| LTA4H, ANOVA: P=1.000 | | | | | | | | | |  |  |  |  | |  | |  |  | |  | |  |  |
| Time | sample  size | | | Mean (Positive Cell Number per Specimen) | | | Mean (Total Cell Number per Specimen) | Mean (percent Positive) | Std.Dev. (Percent Positive |  | | | | | | | | | | | | |  |
| 0 Hour | 6 | | | 0 | | | ND | 0.0 | 0.0 |  |  |  |  |  | |  | | |  | |  | | |
| 6 Hours | 6 | | | 0 | | | ND | 0.0 | 0.0 |  |  |  |  |  | |  | | |  | |  | | |
| Day 1 | 6 | | | 0 | | | ND | 0.0 | 0.0 |  |  |  |  |  | |  | | |  | |  | | |
| Day 2 | 6 | | | 0 | | | ND | 0.0 | 0.0 |  |  |  |  |  | |  | | |  | |  | | |
| Day 4 | 6 | | | 0 | | | ND | 0.0 | 0.0 |  |  |  |  |  | |  | | |  | |  | | |
| Day 7 | 6 | | | 0 | | | ND | 0.0 | 0.0 |  |  |  |  |  | |  | | |  | |  | | |
| Day 10 | 6 | | | 0 | | | ND | 0.0 | 0.0 |  |  |  |  |  | |  | | |  | |  | | |
| Day 14 | 6 | | | 0 | | | ND | 0.0 | 0.0 |  |  |  |  |  | |  | | |  | |  | | |

| **Table S10. Endosteal Cells** | | | | | | | | | | | | | |
| --- | --- | --- | --- | --- | --- | --- | --- | --- | --- | --- | --- | --- | --- |
| COX-1, ANOVA: P<0.001, Holm-Sidak method | | | | | | | | | | | | | |
| Time | sample  size | Mean (Positive Cell Number per Specimen) | Mean (Total Cell Number per Specimen) | Mean (percent Positive) | Std.Dev. (Percent Positive | P value | | | | | | | |
|  |  |  |  |  |  | 0 Hour | 6 Hours | Day 1 | Day 2 | Day 4 | Day 7 | Day 10 | Day 14 |
| 0 Hour | 6 | 201 | 976 | 20.6 | 13.9 | NA |  |  |  |  |  |  |  |
| 6 Hours | 6 | 21 | 528 | 4.0 | 9.7 | **<0.001** | NA |  |  |  |  |  |  |
| Day 1 | 8 | 14 | 783 | 1.8 | 5.1 | **<0.001** | 1 | N/A |  |  |  |  |  |
| Day 2 | 7 | 0 | ND | 0.0 | 0.0 | **<0.001** | 0.996 | 1 | NA |  |  |  |  |
| Day 4 | 8 | 0 | ND | 0.0 | 0.0 | **<0.001** | 0.995 | 1 | 1 | NA |  |  |  |
| Day 7 | 6 | 0 | ND | 0.0 | 0.0 | **<0.001** | 0.996 | 1 | 1 | 1 | NA |  |  |
| Day 10 | 6 | 0 | ND | 0.0 | 0.0 | **<0.001** | 0.993 | 1 | 1 | 1 | 1 | NA |  |
| Day 14 | 6 | 0 | ND | 0.0 | 0.0 | **<0.001** | 0.995 | 1 | 1 | 1 | 1 | 1 | NA |
| COX-2, ANOVA: P<0.001, Holm-Sidak method | | | | | | | | | | | | | |
| Time | sample  size | Mean (Positive Cell Number per Specimen) | Mean (Total Cell Number per Specimen) | Mean (percent Positive) | Std.Dev. (Percent Positive | P value | | | | | | | |
|  |  |  |  |  |  | 0 Hour | 6 Hours | Day 1 | Day 2 | Day 4 | Day 7 | Day 10 | Day 14 |
| 0 Hour | 6 | 326 | 898 | 36.3 | 13.3 | NA |  |  |  |  |  |  |  |
| 6 Hours | 6 | 269 | 614 | 43.8 | 20.4 | 0.944 | NA |  |  |  |  |  |  |
| Day 1 | 4 | 224 | 767 | 29.2 | 13.3 | 0.962 | 0.636 | NA |  |  |  |  |  |
| Day 2 | 4 | 67 | 635 | 10.6 | 4.1 | 0.06 | **0.005** | 0.496 | NA |  |  |  |  |
| Day 4 | 6 | 130 | 578 | 22.5 | 9.5 | 0.628 | 0.106 | 0.927 | 0.839 | NA |  |  |  |
| Day 7 | 6 | 196 | 705 | 27.8 | 10.8 | 0.914 | 0.452 | 0.865 | 0.49 | 0.916 | NA |  |  |
| Day 10 | 6 | 243 | 594 | 40.9 | 10.0 | 0.893 | 0.902 | 0.832 | **0.014** | 0.257 | 0.661 | NA |  |
| Day 14 | 5 | 318 | 630 | 50.4 | 7.3 | 0.647 | 0.942 | 0.253 | **<0.001** | **0.016** | 0.099 | 0.904 | NA |
| 5-LO, ANOVA: P=1.000 | | | | | |  |  |  |  |  |  |  |  |
| Time | sample  size | Mean (Positive Cell Number per Specimen) | Mean (Total Cell Number per Specimen) | Mean (percent Positive) | Std.Dev. (Percent Positive |  | | | | | | | |
| 0 Hour | 6 | 0 | ND | 0.0 | 0.0 |  |  |  |  |  |  |  |  |
| 6 Hours | 6 | 0 | ND | 0.0 | 0.0 |  |  |  |  |  |  |  |  |
| Day 1 | 6 | 0 | ND | 0.0 | 0.0 |  |  |  |  |  |  |  |  |
| Day 2 | 6 | 0 | ND | 0.0 | 0.0 |  |  |  |  |  |  |  |  |
| Day 4 | 6 | 0 | ND | 0.0 | 0.0 |  |  |  |  |  |  |  |  |
| Day 7 | 6 | 0 | ND | 0.0 | 0.0 |  |  |  |  |  |  |  |  |
| Day 10 | 6 | 0 | ND | 0.0 | 0.0 |  |  |  |  |  |  |  |  |
| Day 14 | 6 | 0 | ND | 0.0 | 0.0 |  |  |  |  |  |  |  |  |
| LTA4H, ANOVA: P=1.000 | | | | | |  |  |  |  |  |  |  |  |
| Time | sample  size | Mean (Positive Cell Number per Specimen) | Mean (Total Cell Number per Specimen) | Mean (percent Positive) | Std.Dev. (Percent Positive |  | | | | | | | |
| 0 Hour | 6 | 0 | ND | 0.0 | 0.0 |  |  |  |  |  |  |  |  |
| 6 Hours | 6 | 0 | ND | 0.0 | 0.0 |  |  |  |  |  |  |  |  |
| Day 1 | 6 | 0 | ND | 0.0 | 0.0 |  |  |  |  |  |  |  |  |
| Day 2 | 6 | 0 | ND | 0.0 | 0.0 |  |  |  |  |  |  |  |  |
| Day 4 | 6 | 0 | ND | 0.0 | 0.0 |  |  |  |  |  |  |  |  |
| Day 7 | 6 | 0 | ND | 0.0 | 0.0 |  |  |  |  |  |  |  |  |
| Day 10 | 6 | 0 | ND | 0.0 | 0.0 |  |  |  |  |  |  |  |  |
| Day 14 | 6 | 0 | ND | 0.0 | 0.0 |  |  |  |  |  |  |  |  |

| **Table S11. COX-2 Positive Osteoclasts** | | | | | | | | | |
| --- | --- | --- | --- | --- | --- | --- | --- | --- | --- |
| Internal Callus, ANOVA: P=0.0001, Holm-Sidak method | | | | | | | | | |
| Time | sample  size | Mean (COX-2 Positive Cell Number per 40000 um^2^) | Mean (TRAP Positive Cell Number per 40000 um^2^) | Mean (Percent Positive) | Std.Dev. (Percent Positive | P value | | | |
|  |  |  |  |  |  | Day 4 | Day 7 | Day 10 | Day 14 |
| Day 4 | 6 | 5 | 8 | 56.7 | 32.5 | NA |  |  |  |
| Day 7 | 6 | 16 | 18 | 88.1 | 9.7 | **0.018** | NA |  |  |
| Day 10 | 6 | 14 | 14 | 97.8 | 3.0 | **0.002** | 0.563 | NA |  |
| Day 14 | 6 | 16 | 16 | 98.4 | 1.9 | **0.002** | 0.667 | 0.945 | NA |
| External Callus, ANOVA: P<0.001, Holm-Sidak method | | | | | | | | | |
| Time | sample  size | Mean (COX-2 Positive Cell Number per 40000 um^2^) | Mean (TRAP Positive Cell Number per 40000 um^2^) | Mean (Percent Positive) | Std.Dev. (Percent Positive | P value | | | |
|  |  |  |  |  |  | Day 4 | Day 7 | Day 10 | Day 14 |
| Day 4 | 7 | 0 | ND | 0 | 0 | NA |  |  |  |
| Day 7 | 7 | 11 | 15 | 75.0 | 22.4 | **<0.001** | NA |  |  |
| Day 10 | 6 | 11 | 11 | 98.2 | 4.3 | **<0.001** | **0.004** | NA |  |
| Day 14 | 6 | 18 | 18 | 98.7 | 1.9 | **<0.001** | **0.005** | 0.946 | NA |

| **Table S12. 5-LO Positive Fibroblasts** | | | | | | | | | | | | | |
| --- | --- | --- | --- | --- | --- | --- | --- | --- | --- | --- | --- | --- | --- |
| COX-1, ANOVA: P<0.001, Holm-Sidak method | | | | | | | | | | | | | |
| Time | sample  size | Mean (Positive Cell Number per 5625 um^2^) | Mean (Total Cell Number per 5625 um^2^) | Mean (Percent Positive) | Std.Dev. (Percent Positive | P value | | | | | | | |
|  |  |  |  |  |  | 0 Hour | 6 Hours | Day 1 | Day 2 | Day 4 | Day 7 | Day 10 | Day 14 |
| 0 Hour | 6 | 0 | ND | 0 | 0 | NA |  |  |  |  |  |  |  |
| 6 Hours | 6 | 6 | 11 | 53.7 | 8.7 | **<0.001** | NA |  |  |  |  |  |  |
| Day 1 | 6 | 1 | 5 | 24.7 | 16.1 | 0.307 | 0.164 | N/A |  |  |  |  |  |
| Day 2 | 6 | 1 | 7 | 21.2 | 21.2 | 0.44 | 0.085 | 0.982 | NA |  |  |  |  |
| Day 4 | 6 | 7 | 14 | 49.2 | 26.3 | **<0.001** | 0.989 | 0.299 | 0.191 | NA |  |  |  |
| Day 7 | 6 | 8 | 14 | 57.1 | 28.2 | **<0.001** | 0.934 | 0.083 | **0.036** | 0.992 | NA |  |  |
| Day 10 | 6 | 5 | 16 | 32.2 | 18.7 | 0.084 | 0.449 | 0.99 | 0.96 | 0.703 | 0.311 | NA |  |
| Day 14 | 6 | 6 | 20 | 27.4 | 6.9 | 0.212 | 0.249 | 0.802 | 0.993 | 0.446 | 0.143 | 0.994 | NA |

| **Table S13. TRAP Positive Cells** | | |
| --- | --- | --- |
| **Periosteum** | | |
| Time | sample  size | Mean (Positive Cell Number per Specimen) |
| 0 Hour | 6 | 44 |
| 6 Hours | 7 | 260 |
| Day 1 | 6 | 304 |
| Day 2 | 6 | 14 |
| Day 4 | 6 | 2 |
| Day 7 | 6 | 8 |
| Day 10 | 5 | 23 |
| Day 14 | 5 | 46 |
| **Endosteum** | | |
| Time | sample  size | Mean (Positive Cell Number per Specimen) |
| 0 Hour | 6 | 308 |
| 6 Hours | 6 | 261 |
| Day 1 | 4 | 203 |
| Day 2 | 4 | 62 |
| Day 4 | 6 | 132 |
| Day 7 | 6 | 212 |
| Day 10 | 6 | 235 |
| Day 14 | 5 | 290 |

| **Table S14. Marrow Cells** | | | |
| --- | --- | --- | --- |
| COX-1, ANOVA: P= 0.4 | | | |
| Examiner | Sample Size | Mean (Percent Positive) | StdDev |
| 1 | 6 | 80.8 | 14.1 |
| 2 | 6 | 78.6 | 11.0 |
| 3 | 6 | 70.3 | 15.6 |
| COX-2, ANOVA: P= 0.7 | | | |
| Examiner | Sample Size | Mean (Percent Positive) | StdDev |
| 1 | 7 | 62.8 | 10.3 |
| 2 | 7 | 58.6 | 14.9 |
| 3 | 7 | 63.2 | 10.5 |
| 5-LO, ANOVA: P=0.9 | | | |
| Examiner | Sample Size | Mean (Percent Positive) | StdDev |
| 1 | 6 | 75.5 | 17.1 |
| 2 | 6 | 73.5 | 16.5 |
| 3 | 6 | 77.0 | 17.0 |
| LTA4H, ANOVA: P= 0.76 | | | |
| Examiner | Sample Size | Mean (Percent Positive) | StdDev |
| 1 | 6 | 75.76 | 11.89 |
| 2 | 6 | 70.73 | 15.06 |
| 3 | 6 | 70.27 | 15.56 |

| **Table S15. Chondrocytes** | | | |
| --- | --- | --- | --- |
| COX-1, ANOVA: P= 0.9 | | | |
| Examiner | Sample Size | Mean (Percent Positive) | StdDev |
| 1 | 7 | 73.6 | 15.5 |
| 2 | 7 | 77.6 | 13.1 |
| 3 | 7 | 74.0 | 15.0 |
| COX-2, ANOVA: P= 0.7 | | | |
| Examiner | Sample Size | Mean (Percent Positive) | StdDev |
| 1 | 7 | 82.7 | 7.4 |
| 2 | 7 | 81.5 | 8.9 |
| 3 | 7 | 85.4 | 7.7 |
| 5-LO, ANOVA: P=0.9 | | | |
| Examiner | Sample Size | Mean (Percent Positive) | StdDev |
| 1 | 6 | 35.3 | 16.6 |
| 2 | 6 | 31.8 | 14.9 |
| 3 | 6 | 35.9 | 18.2 |
| LTA4H, ANOVA: P= 0.9 | | | |
| Examiner | Sample Size | Mean (Percent Positive) | StdDev |
| 1 | 5 | 62.3 | 4.5 |
| 2 | 5 | 63.4 | 3.3 |
| 3 | 6 | 62.5 | 2.8 |

| **Table S16. Intramedullary Fracture Site** | | | |
| --- | --- | --- | --- |
| COX-1, ANOVA: P=1.0 | | | |
| Examiner | Sample Size | Mean (Percent Positive) | StdDev |
| 1 | 6 | 3.1 | 7.6 |
| 2 | 6 | 2.3 | 5.6 |
| 3 | 6 | 3.0 | 7.3 |
| COX-2, ANOVA: P= 0.2 | | | |
| Examiner | Sample Size | Mean (Percent Positive) | StdDev |
| 1 | 6 | 9.6 | 8.2 |
| 2 | 6 | 13.0 | 17.2 |
| 3 | 6 | 1.6 | 2.30 |
| 5-LO, ANOVA: P= 1.0 | | | |
| Examiner | Sample Size | Mean (Percent Positive) | StdDev |
| 1 | 6 | 15.6 | 7.5 |
| 2 | 6 | 16.1 | 9.2 |
| 3 | 6 | 14.7 | 7.0 |
| LTA4H, ANOVA: P=1.0 | | | |
| Examiner | Sample Size | Mean (Percent Positive) | StdDev |
| 1 | 6 | 0 | 0 |
| 2 | 6 | 0 | 0 |
| 3 | 6 | 0 | 0 |

| **Table S17. Muscle Interstitial Cells** | | | |
| --- | --- | --- | --- |
| COX-1, ANOVA: P=0.7 | | | |
| Examiner | Sample Size | Mean (Percent Positive) | StdDev |
| 1 | 6 | 3.0 | 7.4 |
| 2 | 5 | 3.5 | 7.8 |
| 3 | 6 | 6.6 | 10.3 |
| COX-2, ANOVA: P=0.8 | | | |
| Examiner | Sample Size | Mean (Percent Positive) | StdDev |
| 1 | 7 | 8.5 | 14.2 |
| 2 | 7 | 6.8 | 10.0 |
| 3 | 7 | 4.6 | 6.6 |
| 5-LO, ANOVA: P= 0.6 | | | |
| Examiner | Sample Size | Mean (Percent Positive) | StdDev |
| 1 | 6 | 41.9 | 12.0 |
| 2 | 5 | 49.5 | 14.6 |
| 3 | 6 | 44.2 | 12.6 |
| LTA4H, ANOVA: P= 0.8 | | | |
| Examiner | Sample Size | Mean (Percent Positive) | StdDev |
| 1 | 4 | 19.0 | 14.3 |
| 2 | 5 | 25.8 | 16.7 |
| 3 | 6 | 23.7 | 15.4 |

| **Table S18. Callus Leukocytes** | | | |
| --- | --- | --- | --- |
| COX-1, ANOVA: P= 0.9 | | | |
| Examiner | Sample Size | Mean (Percent Positive) | StdDev |
| 1 | 3 | 52.3 | 20.4 |
| 2 | 4 | 53.3 | 20.5 |
| 3 | 4 | 57.3 | 22.1 |
| COX-2, ANOVA: P=1.0 | | | |
| Examiner | Sample Size | Mean (Percent Positive) | StdDev |
| 1 | 2 | 38.6 | 23.2 |
| 2 | 2 | 38.0 | 14.5 |
| 3 | 2 | 40.6 | 22.1 |
| 5-LO, ANOVA: P= 0.9 | | | |
| Examiner | Sample Size | Mean (Percent Positive) | StdDev |
| 1 | 3 | 34.7 | 10.5 |
| 2 | 3 | 37.5 | 10.4 |
| 3 | 3 | 34.8 | 12.5 |
| LTA4H, ANOVA: P=0.9 | | | |
| Examiner | Sample Size | Mean (Percent Positive) | StdDev |
| 1 | 3 | 38.4 | 10.4 |
| 2 | 3 | 40.1 | 18.8 |
| 3 | 3 | 43.5 | 6.1 |

| **Table S19. Callus Periosteal Leukocytes** | | | |
| --- | --- | --- | --- |
| COX-1, ANOVA: P= 0.8 | | | |
| Examiner | Sample Size | Mean (Percent Positive) | StdDev |
| 1 | 5 | 76.7 | 13.1 |
| 2 | 5 | 80.0 | 14.2 |
| 3 | 6 | 82.4 | 14.8 |
| COX-2, ANOVA: P= 0.7 | | | |
| Examiner | Sample Size | Mean (Percent Positive) | StdDev |
| 1 | 7 | 54.4 | 27.8 |
| 2 | 5 | 58.0 | 11.9 |
| 3 | 6 | 63.9 | 17.1 |
| 5-LO, ANOVA: P= 0.9 | | | |
| Examiner | Sample Size | Mean (Percent Positive) | StdDev |
| 1 | 6 | 69.1 | 14.9 |
| 2 | 6 | 66.8 | 18.0 |
| 3 | 6 | 70.0 | 13.8 |
| LTA4H, ANOVA: P=0.9 | | | |
| Examiner | Sample Size | Mean (Percent Positive) | StdDev |
| 1 | 6 | 65.0 | 17.7 |
| 2 | 5 | 70.3 | 18.2 |
| 3 | 6 | 68.1 | 18.1 |

| **Table S20. COX-2 Positive Periosteal Cells** | | | |
| --- | --- | --- | --- |
| COX-2, ANOVA: P=0.6 | | | |
| Examiner | Sample Size | Mean (Percent Positive) | StdDev |
| 1 | 6 | 0 | 0 |
| 2 | 6 | 0.8 | 2.1 |
| 3 | 6 | 0.7 | 1.8 |

| **Table S21. COX-2 Positive Endosteal Cells** | | | |
| --- | --- | --- | --- |
| COX-2, ANOVA: P=0.5 | | | |
| Examiner | Sample Size | Mean (Percent Positive) | StdDev |
| 1 | 6 | 32.5 | 6.3 |
| 2 | 6 | 32.6 | 4.7 |
| 3 | 6 | 27.8 | 10.8 |

| **Table S22. COX-2 Positive Osteoclasts** | | | |
| --- | --- | --- | --- |
| Internal Callus, ANOVA: P=0.3 | | | |
| Examiner | Sample Size | Mean (Percent Positive) | StdDev |
| 1 | 6 | 85.3 | 10.9 |
| 2 | 6 | 69.2 | 22.2 |
| 3 | 6 | 88.1 | 9.7 |
| External Callus, ANOVA: P=0.1 | | | |
| Examiner | Sample Size | Mean (Percent Positive) | StdDev |
| 1 | 7 | 73.9 | 25.3 |
| 2 | 7 | 49.8 | 16.1 |
| 3 | 7 | 75.0 | 22.4 |

| **Table S23. 5-LO Positive Fibroblasts** | | | |
| --- | --- | --- | --- |
| 5-LO, ANOVA: P=1.0 | | | |
| Examiner | Sample Size | Mean (Percent Positive) | StdDev |
| 1 | 6 | 54.7 | 26.8 |
| 2 | 6 | 52.5 | 27.9 |
| 3 | 6 | 57.1 | 28.2 |
